# Key Indicator Detection and Authenticity Identification of Beer Based on Near-Infrared Spectroscopy Combined with Multi-Task Feature Extraction

**DOI:** 10.3390/molecules31071083

**Published:** 2026-03-26

**Authors:** Yongshun Wei, Guiqing Xi, Jinming Liu, Yuhao Lu, Chong Tan, Changan Xu, Weite Li

**Affiliations:** 1 College of Information and Electrical Engineering, Heilongjiang Bayi Agricultural University, Daqing 163319, China; weiyongshun@byau.edu.cn (Y.W.); jinmingliu@byau.edu.cn (J.L.); luyuhao@byau.edu.cn (Y.L.); 2 School of Food Engineering, Harbin University of Commerce, Harbin 150076, China; 3 College of Materials and Energy, South China Agricultural University, Guangzhou 510642, China; xuchangan@scau.edu.cn; 4 School of Artificial Intelligence, Chongqing Technology and Business University, Chongqing 400067, China

**Keywords:** beer, near-infrared spectroscopy, multi-task learning, CNN-LSTM-MHA, feature extraction

## Abstract

To address traditional beer detection limitations, this study proposes a rapid NIRS-based method for detecting key indicators and verifying authenticity. Designing Single-task (STL) and Multi-task learning (MTL) strategies, it employs Variable Importance in Projection for wavelength selection. Deep spectral features were extracted utilizing a Multi-Head Attention (MHA)-fused Convolutional Neural Network (CNN-MHA), Long Short-Term Memory (LSTM-MHA), and hybrid CNN-LSTM-MHA networks. To further enhance model performance, the Bayesian Optimization Algorithm globally optimized network hyperparameters in STL, alongside hyperparameters and multi-task loss weights in MTL. Partial least squares regression, support vector machine regression, and partial least squares discriminant analysis models were established using these features. Results indicate that the MTL-based CNN-LSTM-MHA network effectively learns shared features across multiple tasks, significantly improving model generalization. Specifically, the coefficients of determination ($R^{2}$) for alcohol content and original wort concentration in the validation set were 0.996 and 0.997, respectively, with relative root mean square errors (rRMSE) of 2.024% and 2.515%. In the independent test set, the $R^{2}$ were 0.995 and 0.991, with rRMSE of 2.515% and 2.087%, respectively. Furthermore, 100% classification accuracy was achieved across all datasets. This method provides an efficient technical solution for beer market regulation and real-time detection in production processes.

## 1. Introduction

Beer, as an alcoholic beverage with enormous global consumption, holds significant economic value in the industry [1]. Its quality is directly constrained by the strict control of raw material ratios and fermentation processes. In the beer brewing and evaluation system, alcohol content and original wort concentration are the two most core physicochemical indicators. The former determines the flavor intensity and tax classification of the finished product, while the latter reflects the input of malt raw materials and the nutritional potential prior to fermentation [2,3]. Together, they constitute key parameters for evaluating beer grade, mouthfeel, and the maturity of the fermentation process. Meanwhile, different types of beer exhibit significant differences in chemical composition due to variations in raw material ratios, fermentation strains, and process cycles [4]. These differences constitute the material basis for beer classification and authentication.

With the explosive growth of the craft beer market in recent years, the market environment has become increasingly complex [5]. Some unscrupulous merchants exploit information asymmetry by producing non-fermented beer through blending edible alcohol and adding flavorings and pigments to counterfeit legitimate fermented products or by passing off low-cost industrial beer as high-priced craft beer to obtain exorbitant profits. Such behaviors not only severely disrupt market order but also pose potential food safety hazards due to unknown raw material sources [6]. Therefore, establishing an analytical method capable of rapidly and accurately detecting multiple key physicochemical indicators and verifying authenticity has become an urgent need in the fields of brewing quality monitoring and market regulation.

For a long time, standard detection methods in the beer industry have mainly relied on the density bottle method, gas chromatography, and high-performance liquid chromatography [7,8,9]. Although these methods possess high detection accuracy, they often suffer from drawbacks such as cumbersome sample pretreatment, high consumption of chemical reagents, and long analysis cycles, making it difficult to meet the needs of real-time monitoring on modern high-speed production lines and large-scale rapid market screening. In contrast, near-infrared spectroscopy (NIRS) technology has been widely applied in the field of food analysis due to its advantages of being non-destructive, rapid, and capable of simultaneously acquiring multi-component information [10,11]. However, NIRS data possesses typical high-dimensional characteristics, containing hundreds or thousands of wavelength variables, and suffers from severe collinearity and band overlap between wavebands, making it highly susceptible to interference from environmental noise and baseline drift [12]. These “curse of dimensionality” issues and redundant information, if not effectively handled, can severely weaken the predictive performance and robustness of the model [13]. For spectral feature extraction, the field of traditional chemometrics often employs key wavelength selection strategies aimed at eliminating redundant information, reducing data dimensionality, and improving model interpretability. Variable importance in projection (VIP) analysis is a typical representative of such methods [14]. Although these methods can effectively quantify the explanatory power of each spectral variable for the model to screen out key wavebands, they essentially rely on linear assumptions. They struggle to fully capture the complex, non-linear features and deep abstract information prevalent in NIRS data, thereby limiting the generalization performance of models when processing complex information.

With the rise of deep learning technologies, convolutional neural networks (CNNs) and long short-term memory (LSTM) networks have been gradually introduced into spectral analysis [15,16,17]. CNNs, with their powerful local perception capability, can effectively extract spectral feature peaks but are limited by the receptive field size of convolution kernels, struggling to fully mine long-range dependencies between wavelengths. LSTM networks excel at capturing global dependencies of spectra as sequence data, but their sensitivity to local subtle absorption features is relatively weak when processing high-dimensional data. Therefore, to fully leverage the complementary advantages of CNNs in spectral local feature extraction and LSTM networks in global sequence dependency capture, researchers are currently constructing deep feature extraction architectures fusing CNN and LSTM networks to effectively compensate for the deficiencies of single models in feature representation [18,19]. Simultaneously, addressing the characteristic that only specific wavebands in the spectrum contain key information, the multi-head attention mechanism (MHA) has been introduced to achieve more comprehensive and balanced spectral feature analysis [20,21].

Furthermore, constructing an efficient spectral analysis model depends not only on the choice of network architecture but also on the rationality of the modeling strategy. However, the vast majority of current research still employs the single-task learning (STL) strategy. This “divide and conquer” strategy severs the intrinsic connections between detection objects [22]. Ignoring these associations not only leads to low feature extraction efficiency but also limits the generalization ability of the model. To break through this bottleneck, the multi-task learning (MTL) strategy offers a novel approach, namely, inducing the transfer of knowledge by sharing information from related tasks, forcing the model to learn general feature representations that explain multiple target variables [23]. Recent studies have increasingly begun to compare STL and MTL strategies in the field of spectroscopy. For instance, comparative research on predicting hydroponic macronutrients [22] and monitoring meat quality [24] has empirically demonstrated the superiority of MTL over STL. Compared to modeling each task separately, these studies highlight that MTL can effectively utilize the intrinsic chemical correlations and synergistic information among different components. This shared representation not only improves the prediction accuracy of the primary task but also demonstrates superior robustness when dealing with complex matrix interferences [24,25].

Therefore, the primary aim of this study is to develop a rapid and robust analytical method for the simultaneous quantitative detection of key beer indicators and qualitative authenticity verification using near-infrared spectroscopy. To achieve this, we propose a multi-task deep learning framework integrating convolutional neural networks, long short-term memory networks, and multi-head attention mechanisms. This network is specifically designed to extract highly abstract shared spectral features capable of characterizing multiple targets simultaneously, with the learning process adaptively balanced by the Bayesian optimization algorithm (BOA) [26]. These extracted deep features are subsequently coupled with classical chemometric models to accomplish the final regression and classification tasks [27,28,29]. By systematically comparing this strategy with single-task learning and traditional feature extraction methods, this study seeks to demonstrate the superiority of the multi-task framework in providing an efficient technical solution for comprehensive beer quality control.

## 2. Results and Discussion

### 2.1. Data Analysis

Near-infrared spectroscopy (NIRS) signals are essentially a superposition of chemical information from the sample under test and background noise. In addition to effective absorption information reflecting the sample’s intrinsic chemical properties, the spectrum inevitably contains interference signals generated by environmental fluctuations.

**Figure 1:**
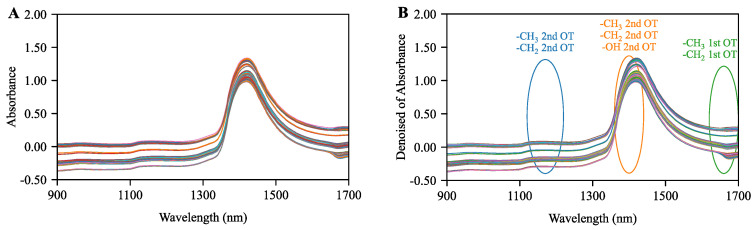

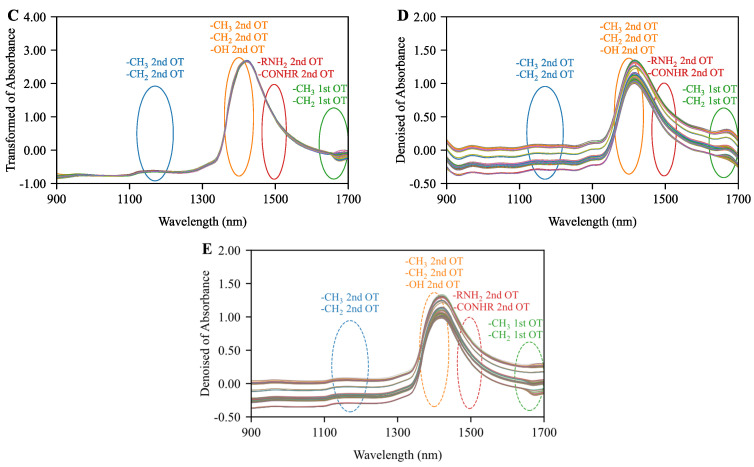
Raw and preprocessed near-infrared spectral data of beer samples. (**A**) Raw spectral data. (**B**) Spectral data preprocessed by wavelet denoising (WD) for alcohol content prediction. (**C**) Spectral data preprocessed by standard normal variate (SNV) for original wort concentration prediction. (**D**) Spectral data preprocessed by a combination of WD and Fourier transform denoising (FTD) for classification authentication. (**E**) Spectral data preprocessed by WD, which was evaluated as the unified optimal method for the multi-task learning (MTL) strategy.

As shown in Figure 1A, the raw spectra were significantly affected by noise and baseline drift. Through comparison and selection, WD was determined as the optimal preprocessing method for the alcohol regression prediction task (results detailed in Table S1). As shown in Figure 1B, WD effectively removed noise while completely retaining the key feature wavebands of ethanol, including the second overtone of -CH3 and -CH2 groups in the 1120~1220 nm band; the second overtone of -CH3, -CH2, and -OH groups in the 1360~1440 nm band; and the first overtone of -CH3 and -CH2 groups in the 1620~1700 nm band [30]. The precise identification of the -CH3, -CH2, and -OH groups is critical because they constitute the fundamental molecular structure of ethanol. Specifically, the overtone absorptions in these spectral regions directly correspond to the fundamental stretching vibrations of the C-H and O-H bonds inherent to alcohol molecules [31]. Preserving these specific vibrational signatures without distortion demonstrates that the selected wavelet denoising method effectively isolates the target chemical information from the baseline drift.

In the original wort concentration regression prediction task, SNV performed best (results detailed in Table S2). As shown in Figure 1C, SNV significantly corrected baseline offset, making the spectral morphology smoother, thereby highlighting feature information in the complex mixed system. Specifically, the 1120~1220 nm band covers the second overtone of -CH2 and -CH3 groups of various sugars; the 1360~1440 nm band covers the second overtone of -OH, -CH2, and -CH3 groups of various sugars and organic acids; the 1463~1530 nm band covers the second overtone of -RNH_2_ groups of amino acids and -CONHR groups of proteins; and the 1620~1700 nm band covers the first overtone of -CH2 and -CH3 groups of various sugars [32]. Highlighting these specific structural groups is highly significant because the original wort is a complex matrix primarily composed of fermentable carbohydrates and nitrogenous nutrients. The prominent absorptions of the -OH, -CH3, and -CH2 groups directly reflect the concentration of various sugars and organic acids constituting the wort carbon skeleton [33]. Meanwhile, the distinct signals from the -RNH2 and -CONHR groups precisely characterize the amino acids and proteins that are essential for subsequent yeast fermentation [34]. By utilizing the SNV method to effectively resolve these highly overlapping signals, our preprocessed spectral data accurately reconstructs the multi-component chemical profile of the wort.

In the classification authentication task, the synergistic combination strategy of WD and FTD yielded optimal results (detailed in Table S3). As shown in Figure 1D, this combined method not only effectively filtered high-frequency random noise and significantly improved the signal-to-noise ratio but also corrected systematic deviations introduced by instruments and the environment in key wavebands such as 1650~1700 nm, greatly enhancing the consistency of spectral data and laying a data foundation for subsequent high-precision classification.

Regarding preprocessing method selection for the MTL strategy, this study used PLSR and PLS-DA models as benchmarks to screen for a globally optimal preprocessing method. The MCPS was used as the evaluation criterion. Through calculation, the WD preprocessing method achieved the minimum comprehensive score of 1.028, indicating that it performed best in balancing multi-task performance; thus, it was selected as the unified preprocessing method for the MTL strategy (results detailed in Table S4), with effects shown in Figure 1E.

Regarding dataset partitioning, the statistical results of the subsets generated by the RS and SPXY algorithms are presented in Table 1. The coefficients of variation for alcohol content and original wort concentration indicators across the three datasets ranged from 22.17% to 39.42%. This result indicates that the distribution characteristics among datasets maintained high consistency and possessed good statistical representativeness, laying a solid data foundation for constructing robust high-performance spectral analysis models.

### 2.2. STL Strategy Spectral Feature Extraction

#### 2.2.1. VIP Feature Variable Selection

**Figure 2:**
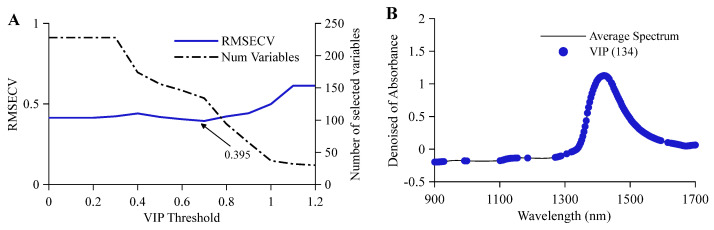

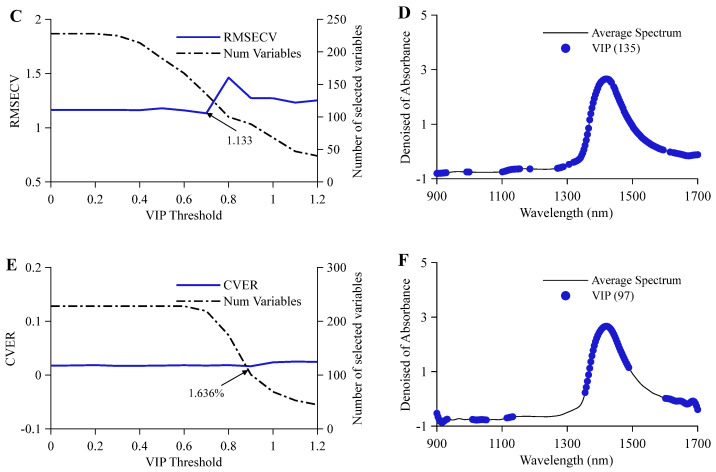
The relationship between VIP wavelength selection and RMSECV, CVER: (**A**,**C**,**E**) represent the optimal VIP thresholds for alcohol content, original wort concentration, and classification identification, respectively. (**B**,**D**) represent the optimal wavelengths for alcohol content and original wort concentration, respectively. (**F**) represents the optimal wavelength for classification identification.

To effectively eliminate redundant information and noise interference in full-spectrum NIRS data, the VIP algorithm was used to construct feature wavelength sets for quantitative detection of alcohol and original wort concentration and for classification authentication of beer samples, respectively. For quantitative detection tasks, the relationship between RMSECV and the number of variables with VIP thresholds was analyzed. As shown in Figure 2A, the RMSECV of the alcohol prediction model showed a trend of gentle decline followed by a slight drop and finally a sharp rise as the threshold increased. When the VIP threshold was set to 0.7, RMSECV reached a minimum of 0.395, retaining 134 feature wavelength variables. As shown in Figure 2B, the optimized wavelength variables were mainly concentrated in the 1350 nm to 1550 nm range; strong absorption peaks in this region corresponded to the first overtone of -OH groups in water and ethanol and the combination band information of -CH groups in beer. As shown in Figure 2C, the RMSECV trend for the original wort concentration prediction model was similar to that of alcohol, achieving a minimum value of 1.133 at a VIP threshold of 0.7, selecting 135 feature wavelength variables. As shown in Figure 2D, the distribution of feature wavelengths highly overlapped with optimized wavelengths for alcohol, indicating related groups between original wort concentration and alcohol, which also reflects the characteristic absorption of hydrogen-containing groups such as -CH and -NH from organic matter like sugars and proteins in original wort in the near-infrared region. For the classification authentication task, the relationship between CVER and the number of wavelength variables with VIP thresholds was established. As shown in Figure 2E, the classification model’s CVER remained relatively stable when the threshold was less than 0.9, reaching a minimum of 1.636% at a threshold of 0.9, after which the error rate rose slightly due to the elimination of key features. Under this optimal threshold, 97 feature wavelength variables were screened. As shown in Figure 2F, compared to full-spectrum data, the VIP algorithm significantly reduced variable dimensionality while retaining key spectral feature information; the optimized wavelengths were mainly distributed in the broad absorption band near 1400 nm, closely related to the comprehensive spectral response of internal components in beer samples.

#### 2.2.2. CNN-MHA Spectral Feature Extraction

This study utilized BOA to iteratively optimize CNN-MHA hyperparameters to achieve optimal extraction of features for different single tasks. As shown in Table 2, for the alcohol regression prediction task, the optimization algorithm matched a convolution kernel combination with sizes of 3, 6, and 23, with 14 filters. This parameter configuration indicates that the network used small convolution kernels of size 3 to precisely capture sharp feature absorption peaks of ethanol, while using large convolution kernels of size 23 to smooth background noise such as baseline drift [35]. On this basis, MHA assigned higher weights to wavebands containing key chemical groups by computing the internal correlation of spectral features in parallel, further suppressing the interference of background noise on model feature expression. An initial learning rate of 0.014209 and an L2 regularization parameter of 1.01930 × 10^−10^ were synchronously matched, indicating that the features required for the alcohol regression prediction task were extremely clear in the spectral data, and the network did not need severe weight penalties to avoid overfitting [36]. For the original wort concentration regression prediction task, the optimization algorithm increased the number of filters to 22 and matched a convolution kernel combination of sizes 4, 7, and 24. More filters and larger convolution kernel sizes aimed to enhance the network’s ability to resolve weak overlapping signals and cover broad-band backgrounds. Notably, the initial learning rate for this task was set to a relatively low 0.003492, combined with an L2 regularization coefficient of 1.02493 × 10^−9^. This low learning rate strategy reflects the more complex optimization landscape faced by the original wort concentration regression prediction task. Since its composition includes various substances, spectral data contains a large number of overlapping group signals, and signal intensity is relatively weak, a lower learning rate helps the network perform refined exploration in a complex search space and prevent getting trapped in local optima [32]. In the beer classification authentication task, the optimized network adopted a convolution kernel combination of sizes 3, 6, and 15, with 15 filters, indicating that a moderate receptive field was sufficient to distinguish key spectral fingerprints of craft and industrial beers.

**Figure 3:**
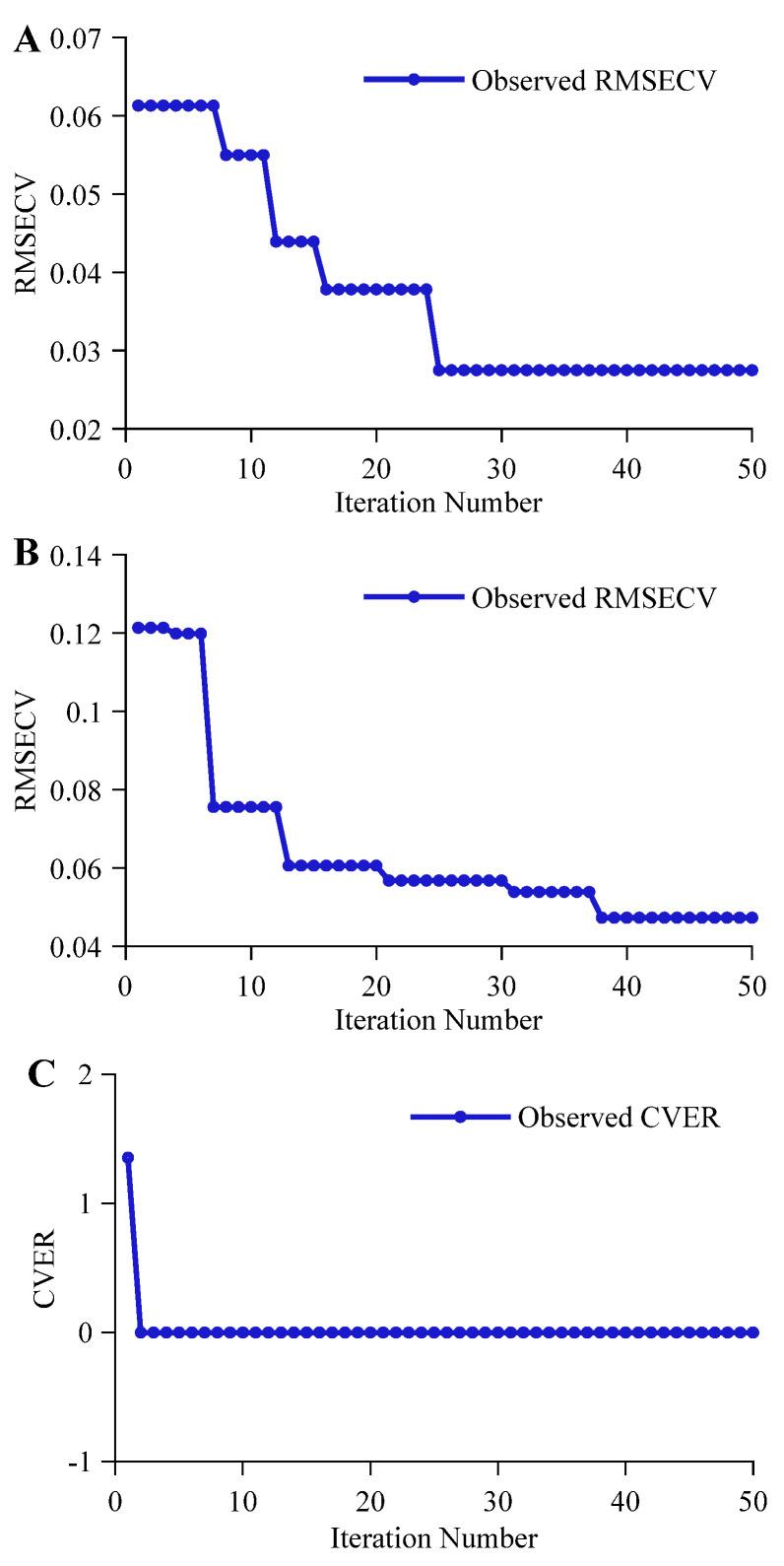
CNN-MHA iterative process based on BOA-optimized STL strategy: (**A**) Alcohol content regression prediction; (**B**) Original wort concentration regression prediction; (**C**) Beer classification and identification. BOA: Bayesian Optimization Algorithm; STL: Single-Task Learning; RMSECV: Root Mean Square Error of Cross-Validation; CVER: Cross-Validation Error Rate.

Looking at the optimization iteration process, there were significant differences across tasks. As shown in Figure 3A, the alcohol regression prediction task achieved the optimal hyperparameter combination at the 25th iteration, with the optimal fitness value RMSECV converging to 0.0275. Its faster convergence speed is mainly attributed to the specific, significant, and clearly composed NIRS absorption features of ethanol in beer, allowing CNN-MHA to quickly lock onto -CH3, -CH2, and -OH groups of ethanol for feature extraction. The optimization iteration process for the original wort concentration regression prediction task is shown in Figure 3B, with the optimal fitness value RMSECV optimizing to 0.0439. Its optimization process showed a stepwise downward trend, and the convergence speed was significantly slower than that of the alcohol task. The main reason may be that the components of original wort concentration include polysaccharides, proteins, and amino acids, and their spectral responses involve numerous overlapping group signals such as -OH, -CH2, -CH3, -RNH_2_, and -CONHR, with relatively weak signal intensities. BOA must constantly probe within a complex search space, progressively stripping away background interference and resolving overlapping spectral signals through different multi-scale convolution kernel combinations. This exploration process led to a stepwise decline pattern and slowed down convergence. As shown in Figure 3C, the CVER in the beer classification authentication task dropped to 0 at the 2nd iteration, with the corresponding ACCCV reaching 100%, reflecting the large distinction in spectral features among different categories of beer.

#### 2.2.3. LSTM-MHA Spectral Feature Extraction

LSTM-MHA focuses on capturing sequence dependencies between spectral wavelengths, and the optimization results of its structural parameters directly reflect the sequence complexity of the task. As shown in Table 3, in the alcohol prediction task, BOA optimized the number of hidden units to 49; this medium-scale memory capacity was sufficient to encode ethanol feature wavebands and their contextual association information. The embedded MHA module dynamically captured global dependencies between different wavelength points within this temporal information, ensuring the model precisely focused on key time steps contributing most to the target variable when processing long-sequence spectra [21]. In terms of training strategy, the model matched a learning rate drop factor of 0.713919 and a dropout rate of 0.114964. A moderate dropout rate helped prevent excessive dependency between neurons, while a slower learning rate decay strategy ensured the model retained sufficient parameter update magnitude in the middle and late stages of training to finely tune the fitting of local ethanol features. In the original wort concentration prediction task, the optimization algorithm significantly increased the number of hidden units to 80. High hidden units endowed the network with stronger memory capacity, enabling it to store and transmit coexisting features of multiple components like sugars and proteins in long sequences [37]. This task matched a learning rate drop factor of 0.839542 and set the dropout rate to 0.159912. A high learning rate drop factor means the learning rate decays slowly during training; this strategy is crucial for handling complex tasks like original wort concentration as it allows the model to maintain high search capability over a long time to cope with optimization difficulties brought by complex multi-component signals. In the classification authentication task, the optimized number of hidden units was 37, indicating that global features required for classification were relatively compact, and effective encoding could be achieved without excessive network capacity. Setting a dropout rate of 0.273842 introduced stronger randomness, effectively preventing the model from overfitting under smaller network capacity and enhancing the robustness of classification boundaries.

**Figure 4:**
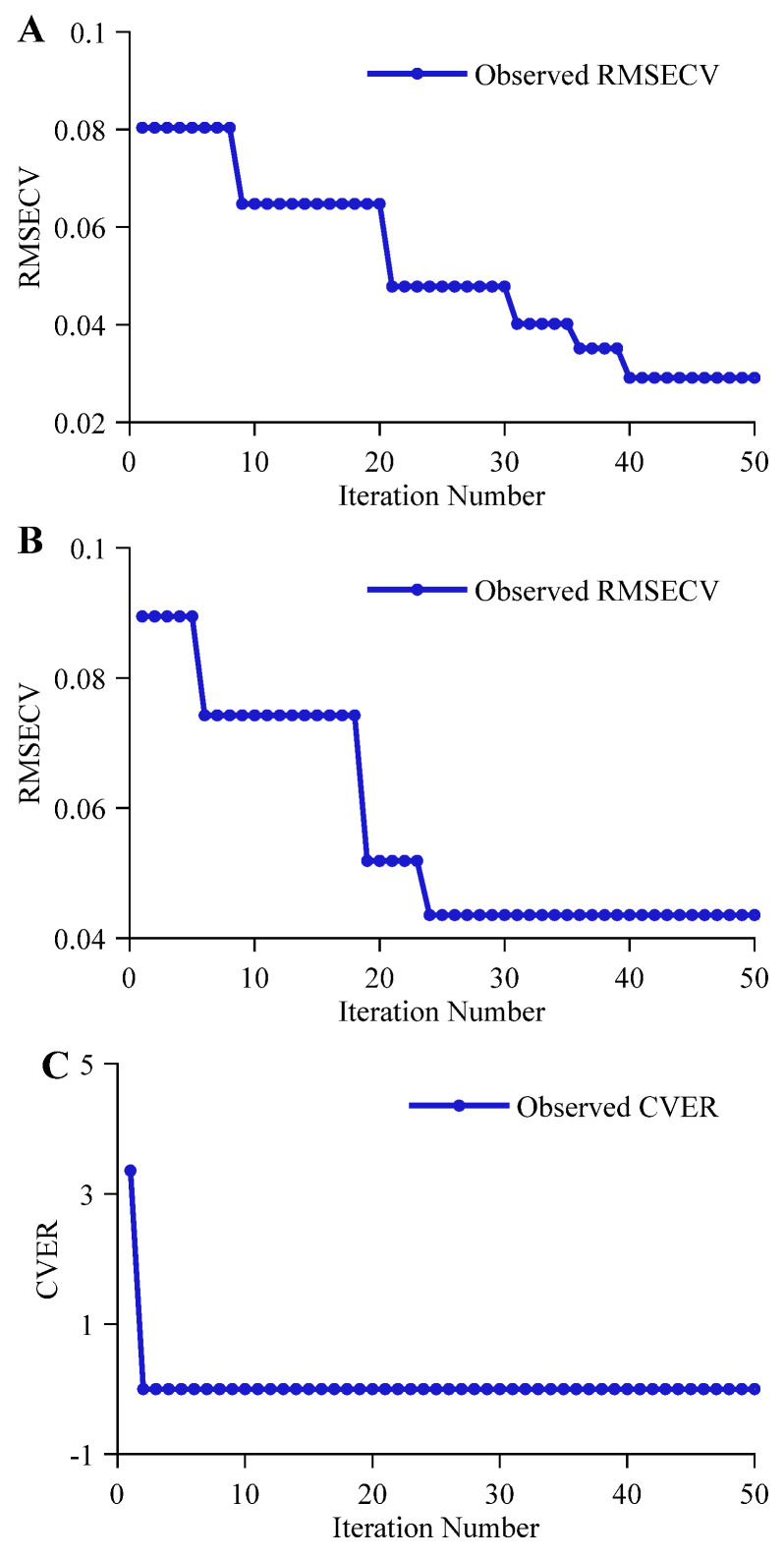
LSTM-MHA iterative process based on BOA-optimized STL strategy: (**A**) Alcohol content regression prediction; (**B**) Original wort concentration regression prediction; (**C**) Beer classification and identification. BOA: Bayesian Optimization Algorithm; STL: Single-Task Learning; RMSECV: Root Mean Square Error of Cross-Validation; CVER: Cross-Validation Error Rate.

The optimization convergence effects of the BOA-optimized LSTM-MHA network for feature extraction varied distinctively across tasks. As shown in Figure 4A, the iterative optimization process for the alcohol regression prediction task presented a progressive convergence form, updating at the 8th, 21st, 31st, 36th, and 40th iterations. The final optimal fitness value RMSECV was 0.0291, slightly higher than the RMSECV value of CNN-MHA under the STL strategy, indicating that for ethanol group signals with obvious local features, the advantage of a single LSTM-MHA network relying on long-term sequence modeling is limited. The convergence curve for the original wort concentration regression prediction task, shown in Figure 4B, presented a significant stepwise downward trend, finally reaching an optimal fitness value RMSECV of 0.0435 after the 24th iteration. For the classification authentication task, the iterative optimization process of the LSTM-MHA network is shown in Figure 4C; LSTM-MHA also updated the CVER value to 0 within 2 optimization iterations, again proving the significant compositional differences among different beers in the classification authentication task.

#### 2.2.4. CNN-LSTM-MHA Spectral Feature Extraction

The CNN-LSTM-MHA network, fusing CNN’s local feature extraction capability, LSTM’s sequence modeling capability, and the MHA mechanism, demonstrated superior feature resolution efficiency and unique training characteristics under the single-task learning strategy. As shown in Table 4, in the alcohol regression prediction task, the optimal small, medium, and large convolution kernels for the CNN-LSTM-MHA network were 3, 7, and 19 respectively, with only 9 filters and 46 hidden units. This streamlined architecture benefited from the effective removal of redundant information by the front-end convolution layers, allowing subsequent sequence layers to process only high signal-to-noise ratio feature maps [18]. Subsequently, MHA secondarily refined the encoded deep features, adaptively adjusting the response intensity of different feature channels, achieving feature integration from local features to global relationship dependencies [20]. Analyzing from a hyperparameter perspective, this model matched a high initial learning rate of 0.049820, but the learning rate drop factor was only 0.243721. This strategy indicates that the network could quickly converge near the optimal solution in the early stages of training, followed by fine-tuning stabilization through a sharply decreasing learning rate. In the original wort concentration regression prediction task, the optimized network used a convolution kernel combination of sizes 4, 6, and 15 and 13 filters, with 32 hidden units. Notably, compared to the 80 hidden units required by the single LSTM-MHA network, the CNN-LSTM-MHA network achieved better performance with only 32 units, greatly reducing computational complexity. This task also adopted an aggressive training strategy, with an initial learning rate reaching 0.061375, combined with a learning rate drop factor of 0.259172, allowing the model to quickly converge to the global minimum. In the classification authentication task, the optimized network was configured with a convolution kernel combination of sizes 4, 7, and 17, with 46 hidden units, reflecting extremely high feature expression efficiency.

**Figure 5:**
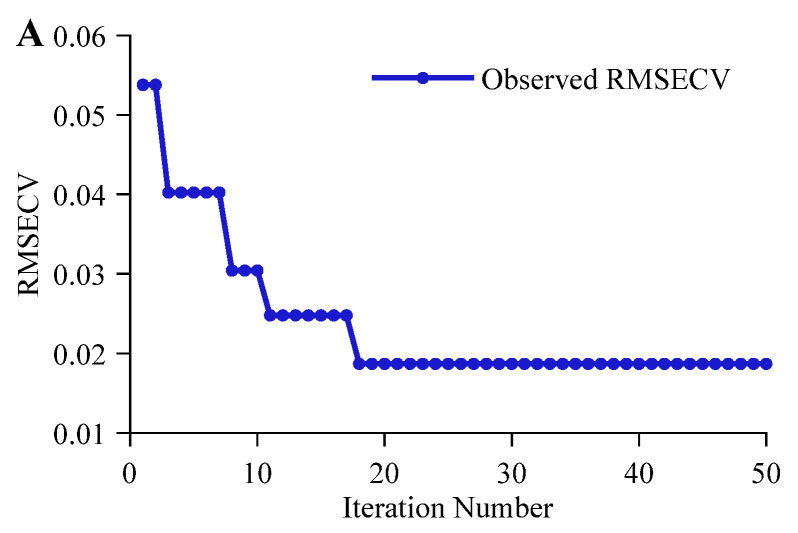

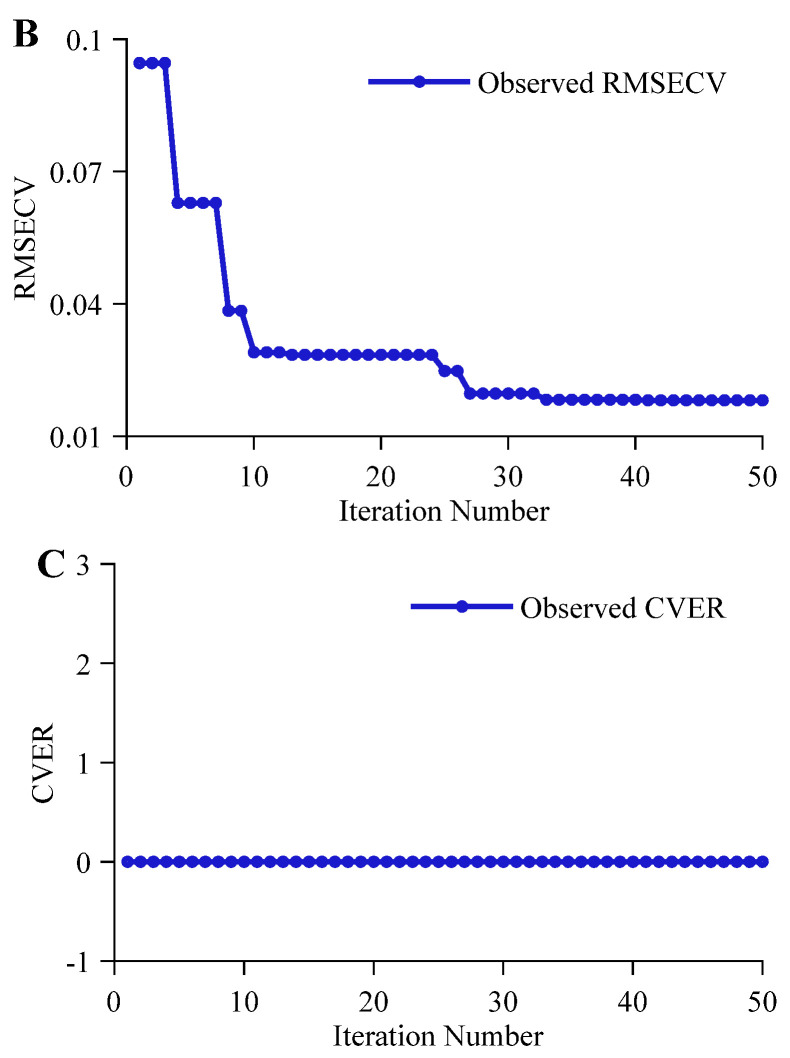
CNN-LSTM-MHA iterative process based on BOA-optimized STL strategy: (**A**) Alcohol content regression prediction; (**B**) Original wort concentration regression prediction; (**C**) Beer classification and identification. BOA: Bayesian Optimization Algorithm; STL: Single-Task Learning; RMSECV: Root Mean Square Error of Cross-Validation; CVER: Cross-Validation Error Rate.

The iterative optimization process of the CNN-LSTM-MHA network in the alcohol regression prediction task is shown in Figure 5A. The model reached the optimal fitness value at the 18th iteration, with an RMSECV of 0.0187, outperforming the other two STL strategy-based networks (CNN-MHA and LSTM-MHA). This indicates that the front-end parallel multi-scale convolution layers effectively filtered noise and extracted ethanol-related local features, enabling the subsequent LSTM-MHA network to focus more on long-term dependencies between these local features [38]. Combined with the multi-head attention mechanism, global deep feature extraction for ethanol spectral signals was further achieved. In the original wort concentration regression prediction task, the iterative optimization process of the CNN-LSTM-MHA network is shown in Figure 5B, also presenting a progressive convergence trend, with a final optimal fitness value RMSECV of 0.0134. Notably, this result was not only significantly lower than the optimal values of single CNN-MHA and LSTM-MHA networks but also converged earlier. This demonstrates that for multi-component complex spectral signals, relying solely on local receptive fields or modeling long-term dependencies alone has limitations. Combining the CNN’s local signal decoupling capability with LSTM’s long-term dependency modeling capability can achieve deeper feature extraction for original wort concentration. For the classification authentication task, the iterative optimization process of the CNN-LSTM-MHA network is shown in Figure 5C, where its CVER dropped to 0 in the first iteration, further proving the effectiveness of the multi-network synergistic mechanism in reinforcing feature extraction.

### 2.3. MTL Strategy Spectral Feature Extraction

The MTL strategy proposed in this study aimed to construct a shared deep feature extraction space, enabling alcohol regression prediction, original wort concentration regression prediction, and classification authentication tasks to synergistically update network parameters and synchronously extract deep features for the three tasks [39]. Unlike the STL strategy, the core of MTL lies in finding a set of optimal task weights while optimizing network hyperparameters via BOA, weighting and fusing the fitness values of the three tasks into a unique global fitness value. This mechanism forces the network to balance the needs of different tasks during the feature extraction stage; that is, features extracted by shared layers must possess both regression fitting capabilities for quantitative indicators and category distinction capabilities for qualitative indicators. This joint optimization process can effectively mine potential chemical associations between different indicators, such as the intrinsic connection between original wort concentration and alcohol content in fermentation mechanisms, thereby using easy-to-learn tasks to assist hard-to-learn tasks and improving feature generalization performance. The hyperparameter and task weight optimization results for different networks under the MTL strategy are shown in Table 5.

For the CNN-MHA network, optimized results showed that its optimal small, medium, and large convolution kernels were set to 5, 9, and 21 respectively, with 14 filters and a feature dimension reaching 190. This gradient convolution kernel combination indicates that the network uses a multi-scale view to resolve spectra: using size 5 small kernels to capture overtone absorption information of sharp -OH or -CH groups, size 9 medium kernels to extract local group information, and size 21 large kernels to cover broad-band background trends. To balance differences among multi-tasks, MHA played a key feature allocation role here, helping shared convolution layers capture common spectral features capable of simultaneously characterizing alcohol regression, original wort regression, and classification tasks, avoiding feature extraction bias towards any single task [40]. The model’s L2 regularization coefficient was only 4.62693 × 10^−7^; this low value combined with an initial learning rate of 0.001006 indicates that the CNN architecture itself, through local connections and weight-sharing mechanisms, already possessed strong anti-overfitting capabilities and maintained robustness without strong external penalties. Regarding task weight allocation, the BOA algorithm set the weights for alcohol regression prediction, original wort concentration regression prediction, and classification authentication to 0.3, 0.5, and 0.2, respectively. This result again proves that original wort concentration corresponds to the sum of sugars, proteins, and various organic substances in wort, with its NIRS response involving multiple severely overlapping absorption bands, making feature extraction most difficult; thus, it was assigned the highest weight to prevent the network from falling into local optima.

For the LSTM-MHA network, although BOA optimized the architecture’s hidden unit count to 103 in an attempt to accommodate more contextual information, the learning rate drop factor was as high as 0.559753, and a smaller L2 regularization coefficient of only 1.37999 × 10^−8^ was used. Without strong constraints, high-dimensional recurrent structures easily fall into rote memorization of training set noise rather than learning essential spectral features [41]. MHA, through parallel attention of multiple subspaces, sought shared dependency patterns of different tasks on spectral sequences, mitigating gradient conflicts between tasks to some extent. In weight allocation, alcohol regression prediction, original wort concentration regression prediction, and classification authentication weights were set to 0.3, 0.4, and 0.3 respectively; this relatively balanced allocation reflects that under this architecture, the importance difference of global sequence features for various tasks was small.

For the CNN-LSTM-MHA network, the Table 5 data shows that optimal small, medium, and large convolution kernels were 2, 11, and 20 respectively, with filters increased to 28, hidden units reaching 140, and feature dimension being 187. This parameter configuration reflects extremely strong feature resolution capability, where size 2 very small kernels could precisely capture extremely tiny fingerprint features, combined with size 20 large kernels summarizing global background, while 140 hidden units ensured the sequence layer had sufficient capacity to simulate long-range dependencies in complex mixed systems. Simultaneously, this hybrid network architecture introduced an L2 regularization coefficient of 1.28982 × 10^−5^, significantly higher than the other two networks, effectively constraining the massive parameter space [42]. In this hybrid network architecture, MHA successfully achieved cross-task dynamic feature attention, locking the focus of feature extraction on high-robustness shared features that satisfy all three tasks simultaneously, thereby significantly accelerating the model’s convergence process. Regarding task weights, the BOA algorithm again selected a combination of 0.3, 0.5, and 0.2, establishing an optimization direction dominated by original wort concentration and assisted by alcohol content, utilizing multi-head attention mechanisms for dynamic weighting to extract a set of highly robust shared deep features.

**Figure 6:**
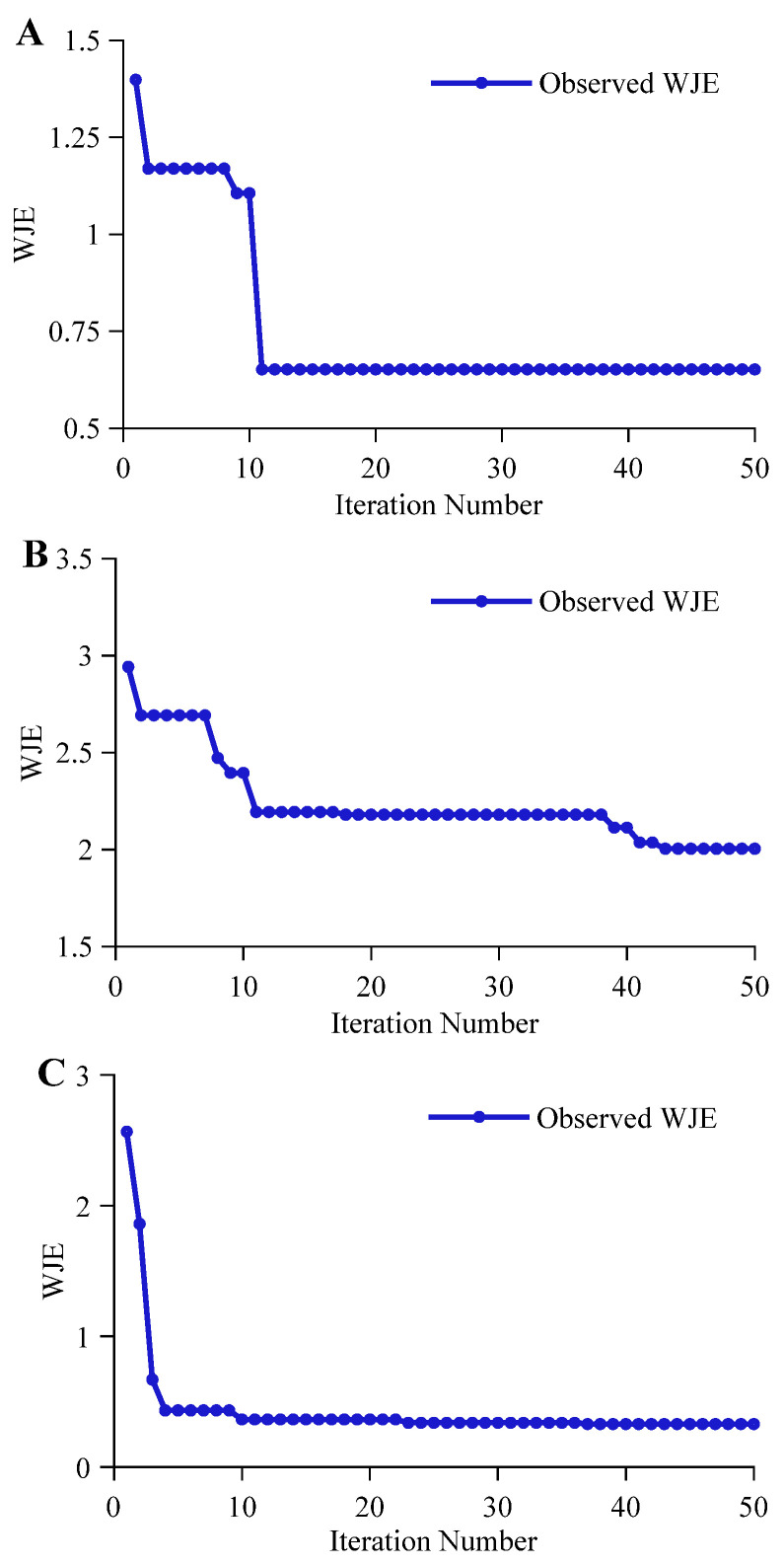
Iterative processes of different feature extraction networks based on the BOA-optimized MTL strategy. (**A**), (**B**), and (**C**) represent the iterative processes of CNN-MHA, LSTM-MHA, and CNN-LSTM-MHA, respectively. BOA: Bayesian Optimization Algorithm; MTL: Multi-Task Learning; WJE: Weighted Joint Error.

Under the multi-task learning strategy, the fitness value (weighted joint error, WJE) of different network architectures showed significant differences in variation with iteration count. Figure 6A displays the iterative process of the CNN-MHA network under the MTL strategy, with its fitness value decline curve showing rapid convergence. A steep downward trend appeared in the early iteration stage, indicating that the network could quickly lock onto common spectral feature regions among different tasks, entering a stable phase at the 10th iteration, with the optimal fitness value WJE finally converging to 0.65171. This efficient convergence mainly benefited from the CNN’s strong perception of local features, enabling it to quickly strip away overlapping group signals highly correlated with original wort concentration regression, alcohol regression, and classification tasks under the guidance of multi-task weights.

As shown in Figure 6B, the iterative optimization process of the LSTM-MHA network under the MTL strategy presented an oscillating downward trend. The optimal fitness value WJE for this architecture was 2.0045, the highest error level among the three compared architectures. The reason for this result may be that LSTM’s unique sequence learning mechanism is unsuitable for simultaneously processing multi-task high-dimensional spectral feature extraction. Since the network struggled to precisely lock onto local absorption positions of specific groups within a single recurrent structure, the algorithm fell into a dilemma when searching for optimal task weight ratios, unable to effectively balance the differentiated demands of regression prediction and classification authentication for feature expression. This structural feature extraction obstacle greatly increased the difficulty of hyperparameter optimization, forcing the model to repeatedly probe within a complex search space without determining a clear gradient optimization direction.

In contrast, the iterative process of the CNN-LSTM-MHA network demonstrated optimal convergence efficiency and stability. As shown in Figure 6C, the network’s optimal fitness value WJE dropped significantly at an extremely fast rate in the early iteration stage, reaching a lower error level in a short time, and subsequently maintained a highly stable convergence state. This trend of rapid decline followed by stable maintenance benefited from the 0.321045 low learning rate drop factor selected by BOA, prompting the network to achieve a rapid switch from global search to local fine-tuning. The effective dimensionality reduction at the front-end convolution layer lightened the burden on the subsequent sequence layer, combined with MHA’s dynamic feature attention, enabling the network to successfully balance gradient contributions of different tasks under the guidance of multi-task weights. Ultimately, the optimal fitness value WJE of this network was as low as 0.32863.

### 2.4. Model Construction and Evaluation

#### 2.4.1. Quantitative Prediction Results and Analysis

Based on features extracted by different feature extraction methods, PLSR and SVM quantitative models were constructed respectively. The modeling performance for quantitative detection of alcohol content and original wort concentration using Full-spectrum (Full), VIP, CNN-MHA, LSTM-MHA, and CNN-LSTM-MHA extracted features was analyzed. The performance of different quantitative prediction models for alcohol content and original wort concentration was evaluated using the $R^{2}$, RMSE, rRMSE, and RPD of calibration, validation, and independent test sets.

As shown in Table 6, in constructing quantitative detection models for beer alcohol content, wavelength variables optimized by VIP were substantially reduced, and the constructed SVM regression model performance was significantly better than full-spectrum modeling, but the constructed PLSR model showed limited performance improvement compared to full-spectrum modeling. This indicates that while VIP effectively eliminated redundant interference and improved the adaptation of feature subsets to SVM non-linear mapping, for linear PLSR models, screening based only on linear correlation might not have significantly changed the expression capability of the original latent variable structure [43]. Compared to traditional feature selection methods, regression models constructed after feature extraction by CNN-MHA and LSTM-MHA based on the STL strategy showed advantages. However, after introducing the MTL strategy, the feature extraction performance of single network architectures showed distinctly different trends. For CNN-MHA, the PLSR model constructed after MTL strategy feature extraction outperformed the STL strategy, but the SVM model performance was lower than the STL strategy. This may be because MTL, through joint training, prompted the network to extract general spectral features with greater robustness and generalization capability; these denoised general features aligned better with PLSR’s linear assumption, thereby improving linear model prediction capability. However, this consideration for multi-tasks also led to the loss of task-specific features for alcohol content, weakening the SVM model’s ability to approximate the optimal solution using refined non-linear features [44]. For LSTM-MHA, both regression models constructed under the MTL strategy performed significantly worse than corresponding models under the STL strategy. This phenomenon indicates a significant negative transfer phenomenon in this network when processing multiple tasks [45]. Due to differences in dependency patterns on spectral sequences across tasks, a single recurrent structure struggled to simultaneously encode sequence patterns of multiple tasks in limited memory units, leading to feature space confusion and reduced expression capability, so the final extracted features satisfied neither linear model regression needs nor non-linear model high-precision mapping.

Specifically, this negative transfer is primarily driven by gradient interference and a lack of local spatial decoupling. During the joint training of MTL, the gradient update directions for the qualitative classification task and quantitative regression tasks frequently conflict. Unlike CNNs, which can isolate and decouple conflicting gradient signals through localized receptive fields, a single LSTM processes the near-infrared spectrum as a holistic continuous sequence. Consequently, forcing the hidden states to simultaneously retain broad qualitative category features and subtle, highly overlapping quantitative features overwhelms its sequence encoding capacity, causing the network to memorize training set noise rather than learning generalizable shared representations.

In stark contrast, CNN-LSTM-MHA demonstrated superior feature integration capabilities by fusing local perception with global sequence modeling. The performance of the PLSR model constructed after feature extraction by the fusion network based on the MTL strategy was slightly lower than the STL strategy, while SVM model performance improved slightly. The reason is that the MTL strategy aims to mine shared latent representations among multiple tasks; features extracted by combining CNN and LSTM possessed extremely high-dimensional non-linearity and high abstractness, which, to some extent, increased the difficulty for linear models to resolve feature space and establish regression relationships, leading to a slight performance drop. Conversely, SVM, with its powerful kernel function mapping capability, could effectively adapt to and resolve these complex non-linear shared features. Meanwhile, considering the prediction accuracy of the SVM model under the STL strategy was already at an extremely high level, the slight performance improvement brought by the MTL strategy may indicate that the prediction accuracy for this indicator was approaching the performance bottleneck under existing data conditions, and the model might have reached a prediction saturation state.

The optimal model for the beer alcohol regression prediction task was the SVM model constructed after deep feature extraction by CNN-LSTM-MHA under the MTL strategy. Its $R^{2}$ for the calibration, validation, and independent test sets was 0.997, 0.996, and 0.995 respectively; the RMSE was 0.104, 0.091, and 0.114; the rRMSE was 2.313%, 2.024%, and 2.515%; and the RPD for the validation and independent test sets was 16.891 and 14.246 respectively. This model performance was significantly superior to various regression models built based on full-spectrum and other feature extraction methods. Its excellent predictive performance can be attributed to ethanol being a major component in beer besides water, with its spectral response possessing extremely high signal intensity and specificity [36]. Driven by the MTL strategy, the CNN-LSTM-MHA network dynamically suppressed strong water absorption interference via MHA, precisely focusing on ethanol group signal regions [21]. Simultaneously, general features learned by the shared layer when processing classification authentication and original wort regression prediction tasks further assisted the model in stripping away background differences. This allowed CNN-LSTM-MHA under the MTL strategy to precisely lock onto key group features and eliminate irrelevant background interference under the guidance of multi-task weights, constructing a regression model that meets the demand for rapid beer alcohol detection.

**Figure 7:**
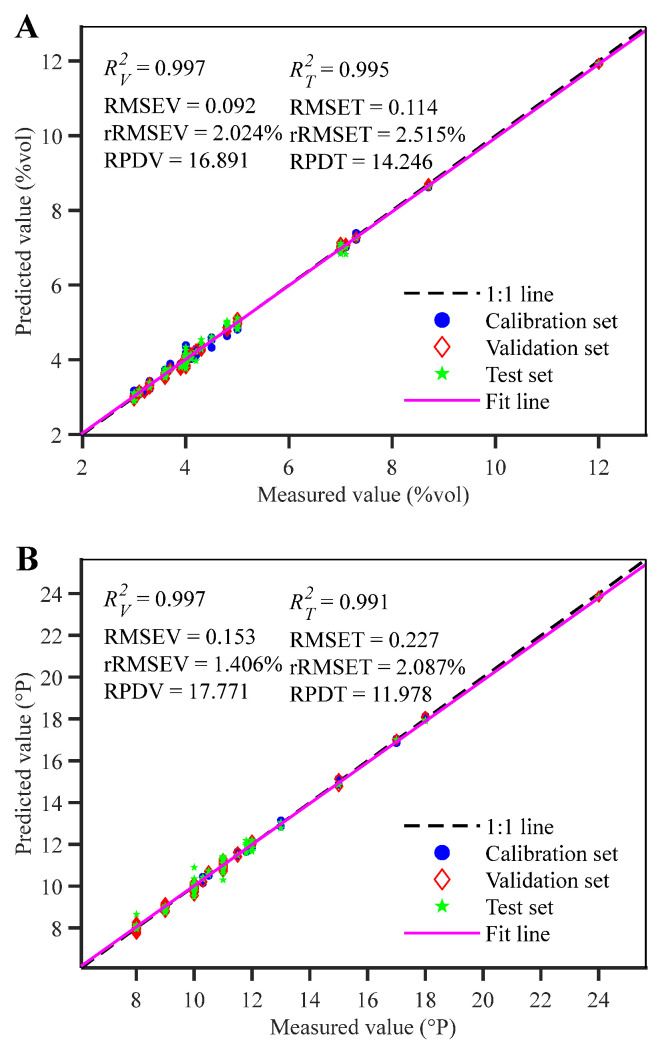
Performance evaluation plot of alcohol content (**A**) and original wort concentration (**B**) models. $R_{c}^{2}$, $R_{v}^{2}$, $R_{t}^{2}$, RMSEC, RMSEV, RMSET, rRMSEC, rRMSEV, and rRMSET represent $R^{2}$, RMSE and rRMSE of calibration, validation and test sets, respectively. RPDV and RPDT represent RPD of validation and test sets.

Looking at the diagonal distribution plot of measured vs. predicted values for beer alcohol content from the CNN-LSTM-MHA+SVM model under the MTL strategy (Figure 7A), the independent test set constructed using the SPXY method showed wide distribution and obvious random characteristics. Sample points for calibration, validation, and independent test sets were basically located near the 1:1 line, and the fitted line basically coincided with the 1:1 line. For unknown samples in the validation and independent test sets, the model showed excellent stability and robustness, further indicating that the CNN-LSTM-MHA network under the MTL strategy could effectively extract deep features from NIRS data, achieving high predictive performance when combined with an adapted regression model. Using the Matlab ttest function to perform a *t*-test on measured and predicted beer alcohol values, the test results returned for both validation and independent test sets were 0, with significance level probabilities of 0.2510 and 0.6552 respectively (both >0.05), and test statistics were less than the critical value *t*_0.05_(83) = 1.989, indicating no significant difference and good consistency between measured and predicted alcohol content values [46].

**Figure 8:**
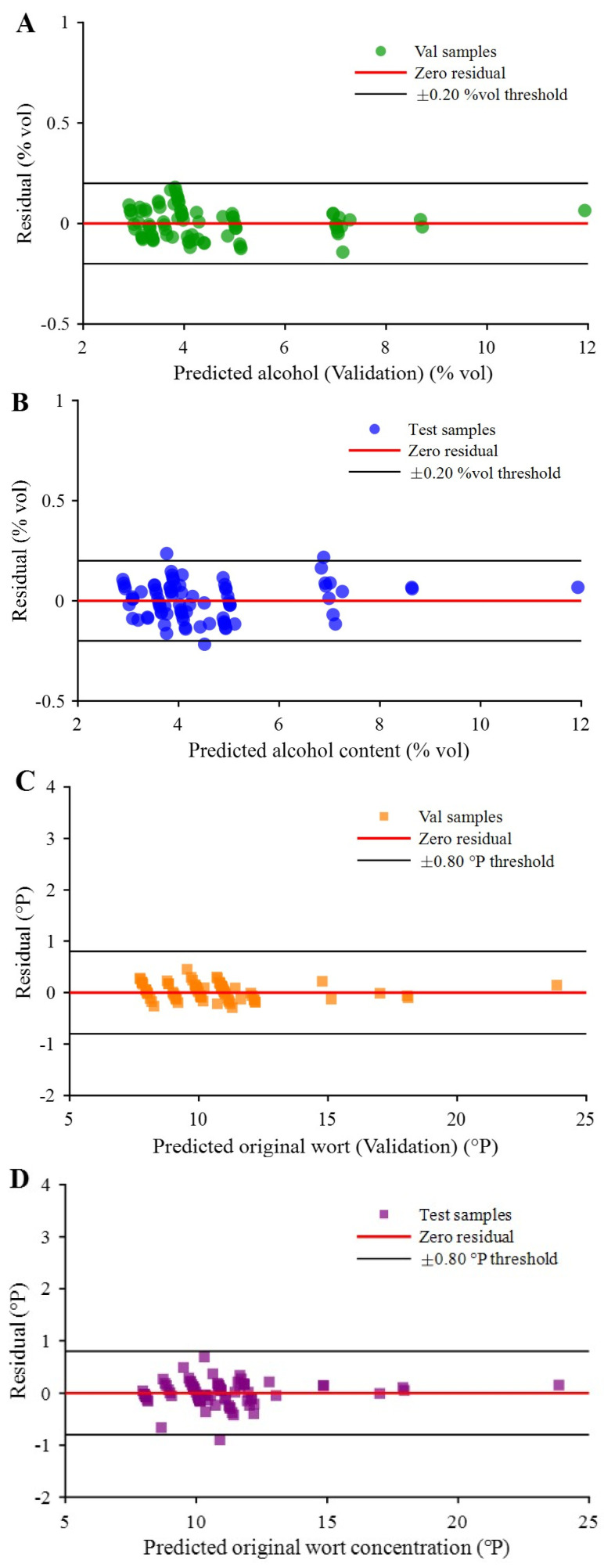
Confidence Interval Plots for Alcohol Content (**A**,**B**) and Original Wort Concentration (**C**,**D**) Models.

To further evaluate the reliability and robustness of the model, this study conducted residual analysis and uncertainty quantification. Confidence interval results for the validation and independent test sets are shown in Figure 8A and Figure 8B respectively. The confidence interval results indicate that residuals for the validation set were distributed between −0.135% vol and 0.184% vol, while the residual range for the independent test set was −0.210% vol to 0.232% vol. Notably, the absolute value of residuals for all samples in the validation set did not exceed 0.20% vol, and 96.4% of samples in the independent test set also met this threshold. These results indicate that the model demonstrated extremely high accuracy and consistency in alcohol content prediction, reflecting good prediction robustness [47].

As shown in Table 7, in constructing quantitative detection models for beer original wort concentration, the performance of the PLSR model constructed after VIP algorithm wavelength selection showed a significant decline compared to full-spectrum modeling. This may be because original wort concentration involves complex components, and VIP lost some key information while eliminating collinear wavelengths, disrupting the integrity of latent variables [48].

In contrast, feature extraction methods based on deep learning showed significant differences, and different network architectures exhibited different variation patterns under STL and MTL strategies. First, for the CNN-MHA network, the MTL strategy demonstrated comprehensive and stable improvement effects. Compared to the STL strategy, the performance of both PLSR and SVM models constructed after feature extraction under the MTL strategy was significantly enhanced. This indicates that for complex indicators containing massive overlapping absorption peaks like original wort concentration, the CNN, with its multi-scale convolution kernel combinations and powerful local perception capability, could effectively utilize shared information among multiple tasks [35]. Through joint training, the network suppressed background noise while extracting general matrix features, making the extracted spectral features not only retain structural information required for linear regression but also reinforce detailed features required for non-linear discrimination, achieving dual performance improvement for linear and non-linear models. However, for the LSTM-MHA network, significant negative transfer occurred under the MTL strategy. Data shows that for the PLSR model constructed after feature extraction by LSTM-MHA under the MTL strategy, validation set performance improved compared to the STL strategy, but independent test set performance declined. Meanwhile, the performance of the SVM model constructed under the MTL strategy was comprehensively weaker than that under the STL strategy. This anomalous phenomenon indicates that when processing long-sequence dependency features of original wort concentration, LSTM was limited by the memory capacity of a single recurrent structure and struggled to simultaneously account for differentiated gradient updates of multiple tasks like alcohol regression prediction and classification authentication. Forced joint multi-tasking led to overfitting on the training and validation sets; although extracted features performed well on the validation set, they lost generalization specificity for original wort concentration on unknown samples, leading to performance decline on the independent test set [49]. Finally, for the CNN-LSTM-MHA network, the comparison between STL and MTL strategies revealed the adaptability differences of linear and non-linear models to deep shared features. Under the MTL strategy, the performance of the PLSR model constructed after feature extraction dropped significantly compared to the STL strategy. This explains that under the MTL framework, through the game and balance of multi-task weights, shared features extracted by the network possessed extremely high abstractness and high non-linearity, exceeding the resolution capability of the linear PLSR model, leading to reduced linear regression performance. However, the situation reversed when employing the non-linear SVM model; the performance of the CNN-LSTM-MHA+SVM model under the MTL strategy increased substantially and far exceeded that under the STL strategy [29].

In summary, regarding feature extraction for the complex indicator of original wort concentration, CNN-LSTM-MHA combined with the MTL strategy demonstrated strong advantages. This advantage stems from the knowledge-sharing mechanism among multi-tasks, where the network utilized features of easy-to-learn tasks like alcohol to assist in learning the complex task of original wort concentration, effectively compensating for STL’s deficiency in resolving complex components. Although these highly abstract shared features sacrificed linear separability, potentially causing PLSR model performance decline, they perfectly matched the kernel function mapping requirements of SVM, fundamentally enhancing feature generalization capability and expression accuracy.

Furthermore, an analysis of the optimal number of latent variables selected for the PLSR models, as presented in Table 6 and Table 7, provides deeper insights into the feature space complexity. Traditional Full-spectrum and VIP-based PLSR models generally required fewer latent variables, typically between 10 and 11. In contrast, models constructed using deep features, particularly those under the MTL strategy, such as LSTM-MHA + PLSR and CNN-LSTM-MHA + PLSR, consistently selected a higher number of latent variables, ranging from 14 to 20. This significant increase in PLS components indicates that the shared representations extracted by the multi-task joint training are highly abstract and contain complex, non-linear multi-task information. Consequently, the linear PLSR algorithm must utilize more principal components to adequately decode this high-dimensional variance and establish regression relationships. This finding structurally corroborates our earlier conclusion: highly abstract shared features sacrifice linear separability, explaining the limited PLSR performance, while perfectly matching the kernel function mapping requirements of SVM.

The optimal regression prediction model for beer original wort concentration was also the SVM model constructed after deep feature extraction by CNN-LSTM-MHA under the MTL strategy. Its $R^{2}$ for the calibration, validation, and independent test sets was 0.998, 0.997, and 0.991 respectively; the RMSE was 0.124, 0.153, and 0.227; the rRMSE was 1.141%, 1.406%, and 2.087%; and the RPD for the validation and independent test sets reached 17.771 and 11.978 respectively. This model performance was significantly superior to regression models constructed based on full-spectrum and other feature extraction methods. Its superior performance is mainly attributed to the MTL strategy cleverly utilizing the intrinsic mechanistic coupling relationship between original wort concentration variation and alcohol content. Through joint optimization of the shared feature space, obvious alcohol features assisted in correcting the feature extraction of the complex original wort concentration [50]. Furthermore, addressing the difficulty that spectral signals of components like polysaccharides and proteins in original wort are weak and easily interfered with by moisture, the CNN-LSTM-MHA architecture, relying on MHA’s dynamic weighting capability, effectively suppressed strong background noise and achieved deep mining of low-signal-intensity key features in complex matrices. This method not only retained local features but also covered global chemical information, achieving feature expression capabilities difficult to realize with traditional feature selection methods and STL strategy feature extraction methods [51].

Looking at the diagonal distribution plot of measured vs. predicted values for original wort concentration from the CNN-LSTM-MHA+SVM model under the MTL strategy (Figure 7B), sample points were tightly distributed near the 1:1 line, and the fitted line highly coincided with the 1:1 line, indicating stable model fitting performance.

Performing a *t*-test on measured and predicted beer original wort concentration values, test results for both validation and independent test sets were 0, with significance level probabilities of 0.0799 and 0.7949 respectively (both >0.05), and test statistics were less than the critical value *t*_0.05_(83) = 1.989, indicating no significant difference between measured and predicted original wort concentrations. Further verification of model uncertainty via residual analysis showed confidence intervals for validation and independent test sets as in Figure 8C,D. Validation set residuals for original wort concentration ranged from −0.294 °P to 0.453 °P, with absolute values for all samples below the set threshold of 0.80 °P. Independent test set residuals were distributed between −0.901 °P and 0.697 °P, with 98.8% of samples meeting the threshold requirement. Results indicate that this model performed excellently in original wort concentration prediction with reasonable residual distribution, possessing high prediction accuracy and stability [52].

#### 2.4.2. Classification Results and Analysis

By constructing PLS-DA models based on various feature extraction methods, the feature extraction capabilities and model performance of full-spectrum data (Full), VIP, and the CNN-MHA, LSTM-MHA, and CNN-LSTM-MHA networks under STL and MTL strategies were deeply analyzed. Performance was evaluated using Accuracy (ACC), Precision, Recall, Macro F1, and Weighted F1 of calibration, validation, and independent test sets.

The performance results of all beer classification authentication models are shown in Table 8. The Full-spectrum PLS-DA model achieved 100% for multiple evaluation metrics in calibration and validation sets; for the independent test set, ACC was 98.809%, Precision was 99.074%, Recall was 98.889%, Macro F1 was 98.965%, and Weighted F1 was 98.808%. The fundamental reason linear models could achieve such excellent baseline performance lies in the essential fingerprint differences in chemical composition among the three types of beer. Craft beer is rich in complex components and spectral information due to all-malt fermentation and heavy hop addition; industrial beer has a relatively simple matrix limited by adjunct addition and standardized processes; while non-fermented beer is mainly blended from alcohol and additives, lacking complex secondary metabolites produced by natural fermentation, resulting in the simplest spectral features [6]. This significant inter-class spectral distinction makes classification tasks easier to distinguish compared to quantitative regression tasks.

After feature wavelength selection using the VIP algorithm, feature dimensionality dropped significantly from 228 to 97, and 100% classification accuracy was achieved on the independent test set. This indicates that the VIP algorithm successfully eliminated redundant wavelengths irrelevant to category determination in the full spectrum and retained the most discriminative core spectral wavelengths. In contrast, deep learning-based feature extraction methods showed deeper advantages in data compression and feature abstraction. Under the STL strategy, CNN-MHA, LSTM-MHA, and CNN-LSTM-MHA networks compressed feature dimensions to 26, 24, and 22 respectively, and all models achieved 100% on all five performance metrics across calibration, validation, and independent test sets. This benefited from the powerful non-linear mapping capabilities of deep neural networks and MHA’s dynamic focusing on key wavebands, enabling models to precisely reconstruct spectral fingerprint differences of different beer categories with extremely low-dimensional deep abstract features [53].

Notably, under the MTL strategy, although the feature dimensions extracted by CNN-MHA, LSTM-MHA, and CNN-LSTM-MHA networks were 190, 143, and 187 respectively—significantly higher than under the STL strategy—their classification performance remained robustly at 100%. This dimensional difference profoundly reveals the operating mechanism of the MTL strategy: in multi-task joint training, the shared feature space must simultaneously carry rich detailed information for high-precision quantitative regression, thus it cannot perform extreme feature compression like the STL strategy for simple classification tasks. However, the perfect accuracy demonstrated by these general deep features containing more information in classification tasks inversely validates the effectiveness of the MTL strategy. Through Knowledge sharing among multiple tasks, the classification task served as an auxiliary constraint, helping the network learn feature expressions that satisfy both high-precision regression prediction and strong discriminative classification, thereby providing an efficient solution for comprehensive beer quality control.

### 2.5. Discussion of Advantages and Limitations

Compared with traditional beer physicochemical indicator detection means, the analysis scheme based on NIRS and MTL proposed in this study demonstrates certain advantages in detection efficiency and integration. Although traditional physicochemical analysis methods have mature accuracy, they are often limited by cumbersome pretreatment steps, reliance on chemical reagents, and long analysis cycles, and usually require different detection equipment for single indicators, making it difficult to meet the needs of modern production lines for simultaneous multi-indicator monitoring. In contrast, this study utilizes the multi-component simultaneous detection advantage of NIRS technology combined with the multi-task learning strategy to achieve simultaneous detection of alcohol content, original wort concentration, and beer authenticity category with just one spectral scan. The laboratory-built online liquid detection platform employed a micro-spectrometer based on DLP technology, significantly reducing hardware deployment costs while enabling in situ, real-time, and non-destructive detection modes by directly coupling fiber probes to production pipelines. More critically, the introduction of multi-task deep learning networks allows single spectral data to be fully mined to serve multiple analysis goals, greatly simplifying the detection process and substantially reducing time costs and labor input for single analyses, providing a highly economically beneficial solution for digital quality control in the beer industry.

Comparing traditional VIP feature selection methods with MTL-based deep feature extraction methods, it can be seen that VIP analysis essentially relies on linear models, focusing on screening wavelength combinations with strong explanatory power for single target variables. Although it can remove some redundant information, its feature expression capability is limited when facing ubiquitous non-linear features and complex overlapping information in near-infrared spectra.

Although our group’s prior research has demonstrated the effectiveness of the CNN-LSTM hybrid network in detecting key beer indicators and authenticating beer authenticity, limitations in the network architecture design prevent the CNN module within this hybrid network from employing multi-scale convolution methods. This hinders its ability to simultaneously capture spectral absorption peak features of varying widths and fails to sufficiently extract critical information from the MHA layer. Furthermore, previous studies primarily focused on single-task independent feature extraction, overlooking the potential intrinsic correlations and synergistic information between alcohol content, original wort concentration, and beer category [52]. In contrast, the proposed MTL strategy overcomes these limitations by integrating a multi-scale CNN, LSTM, and MHA architecture. Through adaptive optimization of network hyperparameters and task weights, the approach compels the network to learn universal deep features capable of representing multiple tasks simultaneously.

Especially for the complex indicator of original wort concentration, the MTL strategy cleverly utilized its strong coupling relationship with alcohol content in fermentation mechanisms, using easily extracted ethanol features to assist in correcting feature resolution of complex components. This knowledge-sharing mechanism endowed the model not only with CNN’s local perception and LSTM’s global sequence modeling capabilities but also achieved cross-task key information focusing through MHA, thereby generating deep shared features with greater robustness and generalization capability than traditional methods.

While the developed hybrid CNN-LSTM-MHA model exhibits higher structural complexity compared to simpler networks like CNN-MHA, this complexity is justified by its superior multi-task integration and robustness. The numerical increase in prediction precision over simpler models might appear marginal because the performance is already approaching the theoretical limit of the dataset. However, the core advantage of the hybrid architecture lies in its ability to construct a single shared feature space that successfully drives three distinct tasks simultaneously without experiencing the negative transfer observed in the standalone LSTM network. The multi-scale convolution effectively decouples complex overlapping signals to reduce the burden on the sequence layer, enabling the model to handle the highly complex matrix of original wort concentration. Therefore, the increased computational cost yields a highly integrated analytical framework, eliminating the need to train and deploy multiple independent models for different brewing indicators.

Although the multi-task deep learning spectral analysis method proposed in this study performed excellently in beer multi-indicator detection, its limitations must be objectively reviewed. First, regarding the data foundation, the reference values for alcohol content and original wort concentration of commercial beer samples in this study directly adopted bottle-label-declared values, while non-fermented beer samples were calculated based on experimental formulas. Although these label values and formula values are within industrial tolerances, there may be slight deviations compared to actual values measured by standard laboratory methods, which, to some extent, limits the evaluation of model prediction accuracy. Future research should introduce standard physicochemical detection data as a standard to further calibrate the model. Second, the sample composition was mainly limited to commercial samples purchased in specific regions and laboratory-simulated samples, failing to fully cover complex beer matrix differences introduced by different brewing processes and special raw materials globally, which may affect the model’s universality when facing extreme or niche beer types. Furthermore, this study found that the MTL strategy is not suitable for all network architectures; for example, the single LSTM-MHA structure exhibited negative transfer under multi-task synergy, and highly abstract non-linear features extracted by MTL led to performance decline in traditional linear models. This indicates that this method has high requirements for network architecture design and regression model adaptability. Subsequent research should be dedicated to expanding the global representativeness of the sample library, further exploring more compatible network architectures, and comprehensively evaluating model robustness in different industrial scenarios through independent external validation sets, solidifying the foundation for practical implementation of the technology.

## 3. Materials and Methods

### 3.1. Experimental Sample Preparation

This study constructed a comprehensive beer dataset containing 336 samples, covering craft beer, industrial beer, and laboratory-prepared non-fermented beer samples.

Commercial Beer Samples: Two categories of commercial beers were purchased from local authorized retailers. Among them, there were 138 craft beer samples, covering 23 different styles from mainstream brands in the market, and 78 industrial beer samples, containing 13 typical styles. To ensure sample representativeness and reduce potential sample differences caused by different production batches, 2 production batches were collected for each style, and 3 independent samples were purchased per batch. The production dates of all samples were controlled within 2 months prior to the collection date to ensure freshness. The reference values for alcohol content (% vol) and original wort concentration (°P) of commercial samples were based on the declared values on the bottle labels (see Table S5 for details).

Non-fermented Beer Samples: To simulate non-fermented beer blended by unscrupulous merchants, this experiment manually prepared similar samples using the control variable method. Raw materials were purchased from local compliant food additive suppliers, including edible alcohol (95% vol), high-purity maltose (99%), food-grade beer flavoring, anhydrous citric acid, and sodium bicarbonate, using distilled water provided by the National Engineering Technology Center for Miscellaneous Grains as the solvent. The experiment included 4 alcohol content gradients of 3% vol, 4% vol, 5% vol, and 7% vol and 5 original wort concentration gradients of 8 °P, 9 °P, 10 °P, 11 °P, and 12 °P. The cross-combination of the two formed 20 different physicochemical indicator ratios, with the proportions of other trace additives remaining constant. Each ratio was prepared 6 times, resulting in 120 non-fermented beer samples. Specific formulation ratios are detailed in Table S6.

Given that bubbles generate light scattering, which significantly interferes with the acquisition accuracy of NIRS, all samples underwent strict degassing treatment before spectral scanning. The experiment used a stirring method to continuously stir the samples after opening until no obvious bubbles overflowed to the naked eye, maximizing the removal of carbon dioxide. Processed samples were immediately transferred to clean, sealed reagent bottles and stored in a cool, dry environment pending measurement.

### 3.2. Spectral Data Acquisition

**Figure 9:**
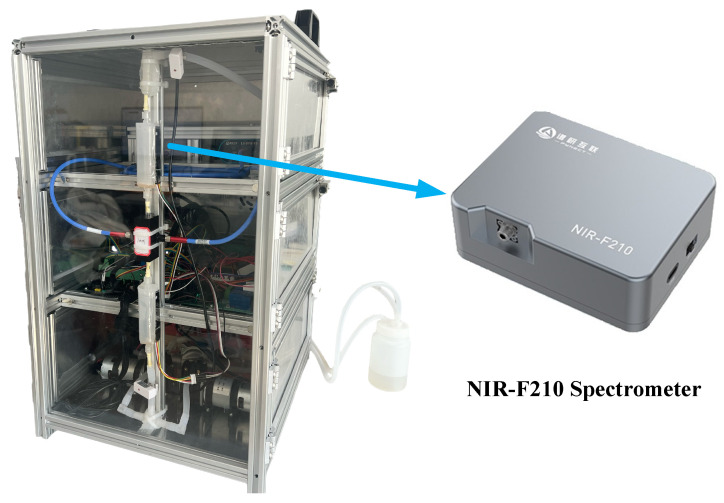
Online liquid proximity infrared detection platform and NIR-F210 micro optical fiber spectrometer.

This study relied on an in-house-built online liquid-phase near-infrared detection platform for data acquisition. The core acquisition equipment employed a NIR-F210 micro-fiber spectrometer (PYNECT, Shenzhen, China) based on digital light processing (DLP) technology (Figure 9). Before formal acquisition, the instrument required a 30 min warm-up to ensure the thermal stability of the light source and spectrometer. The experiment used transmission mode, with a spectral scanning range set to 900~1700 nm, containing 228 wavelength variables. Air was used as the background; to eliminate the influence of environmental fluctuations, the background spectrum was re-acquired every 1 h for baseline correction. For each beer sample, spectral data was scanned continuously 3 times per measurement according to the spectrometer’s fixed settings, and the average value of the 3 scans was taken as the final raw spectrum for that sample to reduce random noise. Following this process, a total of 336 raw spectral data points of beer samples were obtained in this study.

### 3.3. Spectral Preprocessing Methods

Subsequent to data acquisition, employing effective preprocessing methods is essential to suppress random noise and eliminate scattering effects in raw near-infrared spectra [54]. In this study, the performance of spectral preprocessing for beer samples was systematically evaluated using Savitzky-Golay (SG) smoothing, multivariate scattering correction (MSC), standard normal variate (SNV), wavelet denoising (WD), Fourier transform denoising (FTD), and their synergistic combinations.

Based on ten-fold cross-validation, the optimal preprocessing methods for different single-task regression models were determined by comparing the coefficient of determination ($R_{cv}^{2}$), root mean square error of cross-validation (RMSECV), and residual predictive deviation (RPDCV) of the partial least squares regression models. For classification authentication tasks, the optimal methods were selected by comparing the cross-validation accuracy (ACCCV), error rate (CVER), precision, and recall of the partial least squares discriminant analysis models.

Furthermore, to select a globally optimal preprocessing method for the multi-task learning (MTL) strategy, the partial least squares regression and discriminant analysis models were utilized as benchmarks. The multi-task comprehensive performance score (MCPS) was introduced as the evaluation criterion. Considering the significant differences in physical dimensions and numerical magnitudes among the three target indicators, a relative deviation normalization strategy based on optimal values was adopted. The MCPS calculates the ratio of the errors for each task relative to the global minimum error value across all compared preprocessing methods, followed by a weighted sum, as defined in Equation (1):

(1) $$MCPS_{i}=\sum_{j=1}^{3}\omega_{j}\frac{E_{i,j}}{E_{\min,j}}$$

where $MCPS_{i}$ is the comprehensive performance score of the i-th preprocessing method, $\omega_{j}$ represents the weight coefficient of the j-th task (set to 1/3 for each task in this study), $E_{i,j}$ represents the error value of the i-th preprocessing method on the j-th task, and $E_{\min,j}$ represents the minimum error value for the j-th task among all compared preprocessing methods.

A lower MCPS indicates that the comprehensive performance of the preprocessing method across all tasks is closer to the theoretical optimum.

### 3.4. Dataset Partitioning Strategy

To construct quantitative regression and classification models with strong generalization capabilities, a strict dataset partitioning strategy was formulated [55]. Given the limited total sample size, to ensure objectivity in model evaluation, random sampling (RS) was first used to randomly select 84 sample data points from all preprocessed spectral data to serve as an independent test set. This set was strictly isolated to test the applicability of the regression prediction and classification authentication models on unknown samples. Subsequently, to ensure the calibration set could maximally cover the multi-dimensional distribution characteristics of the samples in both spectral and physicochemical property spaces, the sample set partitioning based on the joint X-Y distances (SPXY) algorithm was employed. The SPXY algorithm divided the remaining samples into a calibration set and a validation set at a ratio of 2:1. Ultimately, all data was strictly partitioned into a calibration set, validation set, and independent test set at a ratio of 2:1:1.

### 3.5. Feature Extraction Methods

#### 3.5.1. VIP

VIP analysis quantifies the importance weight of each wavelength variable by analyzing the cumulative explanatory power of independent variables on dependent variables in partial least squares (PLS) models [56]. This study used the full-spectrum data of the calibration set as input. For quantitative regression tasks, VIP calculates the weighted sum of variance explanation of each wavelength for the target variable based on the PLSR model; for classification and authentication tasks, it evaluates the contribution of each wavelength in category separation based on the PLS-DA model. To precisely screen significant features and eliminate interfering background noise, this study set a step size of 0.1 within the threshold range of 0.1 to 1.2. Through 10-fold cross-validation, the optimal VIP threshold was selected using the RMSECV for regression prediction and the CVER for classification authentication as evaluation indicators [57]. Selecting high-scoring wavelength combinations based on this threshold effectively reduces data dimensionality and significantly improves model interpretability and computational efficiency.

#### 3.5.2. CNN-MHA

As a representative of deep feedforward neural networks, CNNs demonstrate superior feature extraction capabilities when dealing with high-dimensional spectral data due to their local receptive field and weight-sharing mechanisms [58]. This study constructed a hybrid architecture deeply fusing a parallel multi-scale CNN and MHA. By configuring parallel branches with three different convolution kernel sizes (small, medium, large), it simultaneously extracts high-frequency details such as sharp absorption peaks and low-frequency trends like baseline drift in spectral data [35]. These multi-scale local features are then fused and input into a dual-head 20-dimensional self-attention module to reinforce correlation features between different wavebands, finally mapping the extracted abstract features to the output space via fully connected layers. In this study, the application of the CNN covered both STL and MTL modes, both using calibration-set full-spectrum data as input. Under the STL strategy, independent CNNs were constructed for alcohol content, original wort concentration, and category authentication, allowing them to focus on extracting features exclusive to specific tasks. Under the MTL strategy, the CNN served as a shared feature extractor aiming to mine general spectral features capable of characterizing multiple indicators simultaneously. However, during the multi-task joint training process, since the fitness values of alcohol regression prediction, original wort concentration regression prediction, and classification authentication tasks differ significantly in physical magnitude, employing fixed or equal weight strategies would easily cause weight updates to be heavily biased towards tasks with high-magnitude losses, thereby neglecting the learning of other tasks [50]. To this end, this study proposed a task weight adaptive joint optimization mechanism based on BOA. The weights of the three tasks were treated as hyperparameters at the same level as network architecture parameters and included in the search space of the BOA. During iterative optimization, BOA dynamically samples weight combinations based on the model’s posterior probability distribution. In this process, to eliminate magnitude influence and prevent high-magnitude tasks from dominating the optimization direction, normalization factors were introduced to standardize the losses of each task. Specifically, the normalization factors for each task were set as the optimal RMSECV or CVER benchmark values obtained by that task under the STL strategy, thereby converting physical errors into relative performance ratios and ensuring fair allocation of multi-task weights. This forces the network to automatically find the optimal weight ratio that balances the gradient contributions of different tasks during the feature extraction stage.

Given that network hyperparameters have a decisive impact on model performance, this study introduced BOA to achieve adaptive hyperparameter optimization. Using calibration-set full-spectrum data as input, the regression task of the STL strategy used 10-fold RMSECV as the fitness value, and the classification task used CVER as the fitness value. In the MTL strategy optimization process, to ensure that the feature extraction architecture optimally adapts to MTL requirements, BOA jointly and adaptively optimized the aforementioned task weights with key hyperparameters of the CNN-MHA network, such as convolution kernel size and number of filters, ensuring the best match between network architecture and task weights. The MTL strategy used the weighted joint error (WJE) calculated by weighting with the optimal weight proportions searched by BOA as the fitness value (Equation (2)). Specific parameter optimization ranges are detailed in Table S7. The CNN under the optimal parameter configuration was used for subsequent spectral feature extraction.

(2) $$WJE=\omega_{alc}\times \frac{RMSECV_{alc}}{N_{alc}}+\omega_{wort}\times \frac{RMSECV_{wort}}{N_{wort}}+\omega_{cls}\times \frac{CVER}{N_{cls}}$$

where $RMSECV_{alc}$ is the RMSECV for the alcohol prediction task, $RMSECV_{wort}$ is the RMSECV for the original wort concentration prediction task, and CVER is the CVER for the classification authentication task. $\omega_{alc}$, $\omega_{wort}$, and $\omega_{cls}$ are the weights searched for the alcohol prediction task, original wort concentration prediction task, and classification authentication task, respectively. $N_{alc}$, $N_{wort}$, and $N_{cls}$ are the normalization factors for the alcohol prediction task, original wort concentration prediction task, and classification authentication task.

#### 3.5.3. LSTM-MHA

Addressing the sequential characteristics of NIRS data, this study employed LSTM networks to capture long-range dependencies and global trends between wavelengths [59]. A sequence feature extraction architecture based on the deep integration of LSTM networks and MHA was constructed. Input spectral data first passed through an LSTM layer for non-linear sequence feature extraction while retaining sequence output patterns and was then projected into a dual-head 20-dimensional self-attention module to capture global dependencies and dynamic weights between features. The feature sequence weighted by the attention mechanism was flattened and finally output deep abstract features through a fully connected layer. Similar to the CNN feature extraction method, the LSTM-MHA network was also applied to both STL and MTL strategies in this study, using calibration set full-spectrum data as input. Under the STL strategy, the LSTM-MHA network independently learned global sequence dependencies for each target variable. Under the MTL strategy, the LSTM-MHA network was trained to capture global sequence dependencies implicit in the spectral sequence that were strongly correlated with both physicochemical indicators and category attributes. To fully unleash the feature extraction capability of the LSTM-MHA network, both strategies employed BOA to iteratively optimize hyperparameters such as the number of hidden layer units, learning rate, and task weights for 50 iterations. The fitness value for the STL strategy was consistent with the CNN-MHA network, and, for the MTL strategy, the task weight adaptive joint optimization mechanism was also adopted, using WJE as the fitness value to achieve synergistic convergence among multi-tasks. Specific parameter optimization ranges are listed in Table S8. The BOA-optimized LSTM-MHA network was used to extract deep sequence features to support subsequent quantitative prediction and classification authentication.

#### 3.5.4. CNN-LSTM-MHA

**Figure 10:**
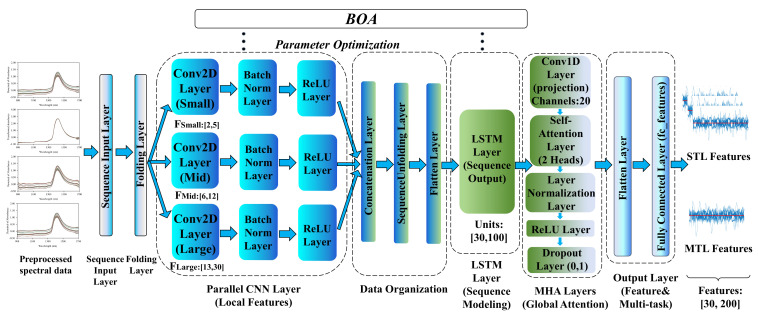
CNN-LSTM-MHA Network Structure.

Addressing the complex characteristics of beer sample NIRS data possessing both local features and global sequence trends, this study constructed a hybrid deep learning architecture integrating parallel CNNs, LSTM networks, and MHA, as shown in Figure 10. First, full-spectrum data entered a parallel convolution module containing three sizes of convolution kernels (small, medium, large) to extract multi-scale local morphological features. Subsequently, these features were flattened and input into a long short-term memory layer for serialization modeling to capture sequence dependencies between wavelengths, compensating for the CNN’s deficiency in global trend modeling. The sequence-encoded information then entered a dual-head 20-dimensional self-attention module to reinforce the influence of key feature wavebands by calculating global correlation weights. Finally, these deeply refined features were integrated and output in the fully connected layer [52].

On this basis, to investigate the impact of different learning strategies on feature generalization capability, this study further compared STL and MTL training strategies, both using calibration-set full-spectrum data as input. Under the STL strategy, the network extracted features separately for alcohol prediction, original wort concentration prediction, and beer classification authentication tasks, aiming to maximize feature specificity for specific tasks. Under the MTL strategy, a parameter sharing mechanism was introduced, enabling the three tasks to share the same CNN-LSTM-MHA feature extractor. Addressing the issue of unbalanced loss functions in MTL, the task weight adaptive joint optimization mechanism was also employed. During iterative optimization, BOA synchronously searched for task weights for alcohol, original wort concentration, and classification authentication alongside key hyperparameters of the CNN-LSTM-MHA network, using WJE as the fitness value. This forced the network to automatically find the optimal combination of weight ratios and network architecture that balanced the gradient contributions of different tasks, thereby achieving synergistic convergence and feature sharing among multiple tasks. Specific parameter optimization ranges for both strategies are detailed in Table S9.

### 3.6. Modeling Methods and Evaluation Metrics

In this study, classical chemometric algorithms, including partial least squares regression (PLSR) [46], support vector machine (SVM) regression [60], and partial least squares discriminant analysis (PLS-DA) [61], were employed as decoupled reference models to evaluate the efficacy of the extracted deep features.

To balance the models’ explanatory power and generalization, robust optimization strategies were implemented. For PLSR and PLS-DA models, Monte Carlo Cross-Validation (MCCV) combined with the minimum predicted residual sum of squares criterion was adopted to determine the optimal number of latent variables (LVs). For the SVM models, grid search combined with 10-fold cross-validation was employed to globally optimize the penalty coefficient and kernel parameters.

To comprehensively evaluate the prediction accuracy and robustness of the models, this study constructed a multi-dimensional evaluation system. For regression prediction tasks, the coefficient of determination ($R^{2}$); root mean square error (RMSE); relative root mean square error (rRMSE) of the calibration, validation, and independent test sets; as well as the residual predictive deviation (RPD) of the validation and independent test sets were used to evaluate regression model performance. When a model’s $R^{2}$ > 0.9, rRMSE < 5%, and RPD > 3.0, this indicates excellent predictive capability. For classification authentication tasks, Accuracy, Precision, Recall, and F1-Score of the calibration, validation, and independent test sets were used [62]. Considering potential sample imbalance, Macro F1 (MF1) and Weighted F1 (WF1) were introduced to provide a more objective evaluation. Higher values for these classification metrics indicate clearer distinction boundaries between different beer categories and more precise and reliable authentication results [52].

All spectral data preprocessing, classical chemometric modeling, deep learning network construction, and Bayesian optimization in this study were performed using MATLAB R2024b.

## 4. Conclusions

This study successfully established a beer multi-indicator simultaneous detection and authentication method based on NIRS combined with MTL, achieving high-precision quantitative detection of alcohol content and original wort concentration and beer authenticity verification. The CNN-LSTM-MHA feature extraction combined with the SVM regression modeling scheme under the MTL strategy yielded optimal performance. Specifically, the alcohol content prediction model $R^{2}$ exceeded 0.99 for both validation and independent test sets, with rRMSE below 2.52%; the original wort concentration prediction model $R^{2}$ exceeded 0.99 for both validation and independent test sets, with rRMSE less than 2.09%. Regarding classification authentication, the model constructed based on shared deep features achieved a 100% three-class accuracy for craft, industrial, and non-fermented beer. The study confirmed that the MTL strategy can utilize correlations between indicators to achieve Knowledge sharing, and its generalization capability is superior to the single-task learning strategy. However, it was also found that negative transfer phenomena occurred under the MTL strategy with a single LSTM network architecture, leading to model performance decline. Through a single-scan, multi-dimensional output analysis mode, this method overcomes the limitations of traditional detection methods being cumbersome and unable to account for multi-dimensional indicators. It demonstrates the superiority and reliability of MTL combined with deep feature extraction in analyzing complex beer matrices, providing a rapid, non-destructive, and efficient solution for quality control and market regulation in the beer industry.

## Figures and Tables

**Table 1:** Alcohol Content and Original Gravity Statistics.

Indicator	Subsets	Number	Mean (% vol/°P)	Max (% vol/°P)	Min (% vol/°P)	Standard Deviation (% vol/°P)	Coefficient of Variation (%)
Alcohol content	Calibration set	168	4.497	12	3	1.773	39.426
	Validation set	84	4.495	12	3	1.537	34.193
	Test set	84	4.532	12	3	1.624	35.834
Original wort	Calibration set	168	10.866	24	8	2.812	25.878
	Validation set	84	10.581	24	8	2.502	23.646
	Test set	84	10.910	24	8	2.419	22.172

**Table 2:** CNN-MHA Parameter Optimization Results.

Target Task	Parameter	Result
Alcohol content (STL)	F_Small	3
	F_Mid	6
	F_Large	23
	NumFilters	14
	FeatureDimension	83
	InitialLearnRate	0.014209
	L2Regularization	1.01930 × 10^−10^
	Parameter count	795,514
Original wort (STL)	F_Small	4
	F_Mid	7
	F_Large	24
	NumFilters	22
	FeatureDimension	113
	InitialLearnRate	0.003492
	L2Regularization	1.02493 × 10^−9^
	Parameter count	1,311,329
Classification and Identification (STL)	F_Small	3
	F_Mid	6
	F_Large	15
	NumFilters	15
	FeatureDimension	26
	InitialLearnRate	0.023671
	L2Regularization	1.00270 × 10^−10^
	Parameter count	418,437

F_Small, F_Mid, F_Large, NumFilters, NumResponses, InitialLearnRate, Alcohol Content Weighting, Original Wort Weight, Classification Discrimination Weight, and L2Regularization represent the small kernel size, medium kernel size, large kernel size, number of filters, feature dimension of the fully connected layer, initial learning rate, alcohol content weight, original wort concentration weight, classification discrimination weight, and L2 regularization coefficient, respectively.

**Table 3:** LSTM-MHA Parameter Optimization Results.

Target Task	Parameter	Result
Alcohol content (STL)	LstmHiddenUnits	49
	FeatureDimension	81
	InitialLearnRate	0.005392
	LearnRateDropFactor	0.713919
	L2Regularization	1.32020 × 10^−9^
	DropoutRate	0.114964
	Parameter count	68,957
Original wort (STL)	LstmHiddenUnits	80
	FeatureDimension	117
	InitialLearnRate	0.013632
	LearnRateDropFactor	0.839542
	L2Regularization	1.69137 × 10^−10^
	DropoutRate	0.159912
	Parameter count	219,389
Classification and Identification (STL)	LstmHiddenUnits	37
	FeatureDimension	24
	InitialLearnRate	0.004821
	LearnRateDropFactor	0.758311
	L2Regularization	1.36281 × 10^−8^
	DropoutRate	0.273842
	Parameter count	33,291

LSTM_Hidden_Units, Feature_Dimension, Initial_LearnRate, LearnRate_DropFactor, L2_Regularization, Dropout_Rate, and Parameter_Count represent the number of hidden layer units in the LSTM, the feature dimension of the fully connected layer, the initial learning rate, the learning rate decay factor, the L2 regularization coefficient, the dropout rate, and the total number of updated variables, respectively.

**Table 4:** CNN-LSTM-MHA Parameter Optimization Results.

Target Task	Parameter	Result
Alcohol content (STL)	F_Small	3
	F_Mid	7
	F_Large	19
	NumFilters	9
	LstmHiddenUnits	46
	FeatureDimension	95
	InitialLearnRate	0.049820
	LearnRateDropFactor	0.243721
	L2Regularization	7.64765 × 10^−5^
	DropoutRate	0.163498
	Parameter count	642,876
Original wort (STL)	F_Small	4
	F_Mid	6
	F_Large	15
	NumFilters	13
	LstmHiddenUnits	32
	FeatureDimension	77
	InitialLearnRate	0.061375
	LearnRateDropFactor	0.259172
	L2Regularization	5.43341 × 10^−7^
	DropoutRate	0.143775
	Parameter count	797,455
Classification and Identification (STL)	F_Small	4
	F_Mid	7
	F_Large	17
	NumFilters	10
	LstmHiddenUnits	46
	FeatureDimension	22
	InitialLearnRate	0.037815
	LearnRateDropFactor	0.319774
	L2Regularization	6.55195 × 10^−7^
	DropoutRate	0.285534
	Parameter count	347,314

F_Small, F_Mid, F_Large, NumFilters, LstmHiddenUnits, FeatureDimension, InitialLearnRate, LearnRateDropFactor, L2Regularization, Alcohol Content Weighting, Original Wort Weight, Classification Discrimination Weight, and DropoutRate represent the small kernel size, medium kernel size, large kernel size, number of filters, number of LSTM hidden units, feature dimension of the fully connected layer, initial learning rate, learning rate drop factor, L2 regularization coefficient, alcohol content weight, original wort concentration weight, classification discrimination weight, and dropout rate, respectively.

**Table 5:** Parameter optimization results of different networks based on the MTL strategy.

Multitask Learning Feature Extraction Method	Parameter	Result
CNN-MHA (MTL)	F_Small	5
	F_Mid	9
	F_Large	21
	NumFilters	14
	FeatureDimension	190
	InitialLearnRate	0.001006
	L2Regularization	4.62693 × 10^−7^
	Alcohol Content Weighting	0.3
	Original Wort Weight	0.5
	Classification Discrimination Weight	0.2
	Parameter count	1,129,247
LSTM-MHA (MTL)	LstmHiddenUnits	103
	FeatureDimension	143
	InitialLearnRate	0.002352
	LearnRateDropFactor	0.559753
	L2Regularization	1.37999 × 10^−8^
	DropoutRate	0.201348
	Alcohol Content Weighting	0.3
	Original Wort Weight	0.4
	Classification Discrimination Weight	0.3
	Parameter count	323.288
CNN-LSTM-MHA (MTL)	F_Small	2
	F_Mid	11
	F_Large	20
	NumFilters	28
	LstmHiddenUnits	140
	FeatureDimension	187
	InitialLearnRate	0.003872
	LearnRateDropFactor	0.321045
	L2Regularization	1.28982 × 10^−5^
	DropoutRate	0.205489
	Alcohol Content Weighting	0.3
	Original Wort Weight	0.5
	Classification Discrimination Weight	0.2
	Parameter count	357,589

**Table 6:** Evaluation Criteria for Quantitative Models of Alcohol Content.

Method	Model	Dimension	$R_{c}^{2}$	$R_{v}^{2}$	$R_{t}^{2}$	RMSEC	RMSEV	RMSET	rRMSEC	rRMSEV	rRMSET	RPDV	RPDT	LVs
STL	CNN-MHA	83	0.981	0.972	0.959	0.244	0.257	0.319	5.426	5.717	7.039	5.981	5.091	N/A
	LSTM-MHA	81	0.977	0.967	0.957	0.269	0.279	0.320	5.982	6.207	7.061	5.509	5.075	N/A
	Full+PLS	228	0.964	0.950	0.947	0.334	0.361	0.353	7.420	8.039	7.783	4.559	4.337	10
	VIP+PLS	134	0.967	0.953	0.951	0.323	0.352	0.340	7.183	7.831	7.502	4.666	4.776	10
	CNN-MHA+PLS	83	0.974	0.965	0.956	0.288	0.303	0.322	6.404	6.741	7.105	5.073	5.043	11
	LSTM-MHA+PLS	81	0.977	0.966	0.958	0.264	0.301	0.315	5.871	6.696	6.951	5.106	5.156	13
	CNN-LSTM-MHA+PLS	95	0.990	0.992	0.983	0.181	0.149	0.207	4.025	3.315	4.568	10.315	7.845	12
	Full+SVM	228	0.980	0.978	0.971	0.252	0.238	0.260	5.604	5.295	5.737	6.458	6.246	N/A
	VIP+SVM	134	0.996	0.996	0.992	0.129	0.121	0.136	2.869	2.692	3.001	12.702	11.941	N/A
	CNN-MHA+SVM	83	0.996	0.997	0.992	0.121	0.115	0.141	2.691	2.558	3.111	13.365	11.518	N/A
	LSTM-MHA+SVM	81	0.997	0.997	0.994	0.107	0.103	0.127	2.379	2.291	2.802	14.922	12.787	N/A
	CNN-LSTM-MHA+SVM	95	0.997	0.997	0.995	0.105	0.092	0.115	2.322	2.047	2.523	16.657	14.001	N/A
MTL	CNN-MHA+PLS	190	0.981	0.980	0.971	0.252	0.239	0.262	5.604	5.317	5.781	6.431	6.198	14
	LSTM-MHA+PLS	143	0.968	0.973	0.942	0.315	0.262	0.367	7.005	5.829	8.098	5.866	4.425	14
	CNN-LSTM-MHA+PLS	187	0.987	0.991	0.977	0.213	0.171	0.246	4.736	3.804	5.428	8.988	6.602	16
	CNN-MHA+SVM	190	0.996	0.996	0.990	0.117	0.103	0.152	2.602	2.291	3.354	14.922	10.684	N/A
	LSTM-MHA+SVM	143	0.988	0.995	0.989	0.192	0.115	0.163	4.270	2.558	3.597	13.365	9.963	N/A
	CNN-LSTM-MHA+SVM	187	0.997	0.996	0.995	0.104	0.091	0.114	2.313	2.024	2.515	16.891	14.246	N/A

$R_{c}^{2}$, $R_{v}^{2}$, $R_{t}^{2}$, RMSEC, RMSEV, RMSET, rRMSEC, rRMSEV, and rRMSET represent $R^{2}$, RMSE and rRMSE of calibration, validation and test sets, respectively. RPDV and RPDT represent RPD of validation and test sets. LVs are the principal components extracted from PLSR, used to optimize model predictions. The optimal number of LVs is determined by the minimum PRESS value obtained during cross-validation.

**Table 7:** Quantitative Model Evaluation Indicators for Original Wort Concentration.

Strategy	Model	Dimension	$R_{c}^{2}$	$R_{v}^{2}$	$R_{t}^{2}$	RMSEC	RMSEV	RMSET	rRMSEC	rRMSEV	rRMSET	RPDV	RPDT	LVs
STL	CNN-MHA	113	0.975	0.973	0.962	0.438	0.429	0.470	4.031	4.054	4.308	5.832	5.147	N/A
	LSTM-MHA	117	0.977	0.970	0.960	0.428	0.448	0.482	3.939	4.234	4.418	5.585	5.019	N/A
	Full+PLS	228	0.907	0.864	0.853	0.819	0.876	1.059	7.564	8.181	9.757	2.712	2.608	11
	VIP+PLS	135	0.898	0.828	0.809	0.895	1.079	1.054	8.237	10.198	9.661	2.319	2.295	10
	CNN-MHA+PLS	113	0.976	0.972	0.963	0.434	0.435	0.464	3.994	4.111	4.253	5.752	5.213	12
	LSTM-MHA+PLS	127	0.976	0.970	0.961	0.433	0.451	0.476	3.985	4.262	4.363	5.548	5.082	11
	CNN-LSTM-MHA+PLS	77	0.988	0.985	0.980	0.307	0.319	0.341	2.825	3.015	3.126	7.843	7.094	13
	Full+SVM	228	0.996	0.993	0.981	0.147	0.220	0.351	1.353	2.079	3.217	11.373	6.892	N/A
	VIP+SVM	135	0.997	0.991	0.984	0.153	0.247	0.305	1.408	2.334	2.796	10.130	7.931	N/A
	CNN-MHA+SVM	113	0.994	0.993	0.973	0.217	0.218	0.396	1.997	2.060	3.630	11.477	6.109	N/A
	LSTM-MHA+SVM	117	0.993	0.992	0.969	0.234	0.233	0.425	2.154	2.202	3.896	10.738	5.692	N/A
	CNN-LSTM-MHA+SVM	77	0.997	0.994	0.977	0.153	0.202	0.366	1.408	1.909	3.355	12.386	6.609	N/A
MTL	CNN-MHA+PLS	190	0.978	0.983	0.971	0.367	0.369	0.412	3.378	3.487	3.776	6.780	5.871	16
	LSTM-MHA+PLS	143	0.976	0.983	0.951	0.433	0.320	0.533	3.985	3.024	4.885	7.819	4.538	20
	CNN-LSTM-MHA+PLS	187	0.986	0.983	0.971	0.349	0.366	0.411	3.212	3.459	3.767	6.836	5.886	15
	CNN-MHA+SVM	190	0.998	0.997	0.986	0.135	0.164	0.288	1.242	1.550	2.640	15.256	8.399	N/A
	LSTM-MHA+SVM	143	0.982	0.991	0.948	0.375	0.232	0.547	3.451	2.193	5.014	10.784	4.422	N/A
	CNN-LSTM-MHA+SVM	187	0.998	0.997	0.991	0.124	0.153	0.228	1.141	1.406	2.087	17.771	11.978	N/A

$R_{c}^{2}$, $R_{v}^{2}$, $R_{t}^{2}$, RMSEC, RMSEV, RMSET, rRMSEC, rRMSEV, and rRMSET represent $R^{2}$, RMSE and rRMSE of calibration, validation and test sets, respectively. RPDV and RPDT represent RPD of validation and test sets. LVs are the principal components extracted from PLSR, used to optimize model predictions. The optimal number of LVs is determined by the minimum PRESS value obtained during cross-validation.

**Table 8:** Evaluation Metrics for Beer Authenticity Classification Model.

Strategy	STL					MTL		
Method	Full	VIP	CNN-MHA	LSTM-MHA	CNN-LSTM-MHA	CNN-MHA	LSTM-MHA	CNN-LSTM-MHA
Dimension	228	97	26	24	22	190	143	187
ACC_C_	100	100	100	100	100	100	100	100
ACC_V_	100	100	100	100	100	100	100	100
ACC_T_	98.809	100	100	100	100	100	100	100
Precision_C_	100	100	100	100	100	100	100	100
Precision_V_	100	100	100	100	100	100	100	100
Precision_T_	99.074	100	100	100	100	100	100	100
Recall_C_	100	100	100	100	100	100	100	100
Recall_V_	100	100	100	100	100	100	100	100
Recall_T_	98.889	100	100	100	100	100	100	100
Macro F1_C_	100	100	100	100	100	100	100	100
Macro F1_V_	100	100	100	100	100	100	100	100
Macro F1_V_	98.965	100	100	100	100	100	100	100
Weighted F1_C_	100	100	100	100	100	100	100	100
Weighted F1_V_	100	100	100	100	100	100	100	100
Weighted F1_T_	98.808	100	100	100	100	100	100	100
LVs	18	18	13	19	19	15	18	17

ACC_C_, ACC_V_, ACC_T_, Precision_C_, Precision_V_, Precision_T_, Recall_C_, Recall_V_, Recall_T_, Macro F1_C_, Macro F1_V_, Macro F1_T_, Weighted F1_C_, Weighted F1_V_, and Weighted F1_T_ represent Accuracy rate, Precision, Recall rate, macro-averaged F1 Score and weighted-averaged F1 Score of calibration, validation and test sets, respectively. LVs are the principal components extracted in PLS-DA, used to optimize model prediction. The optimal number is determined by the minimum ACC obtained through cross-validation.

## Data Availability

Data will be made available upon request.

## Supplementary Materials

The following supporting information can be downloaded at: https://www.mdpi.com/article/10.3390/molecules31071083/s1, Table S1 Results of Different Spectral Preprocessing Methods for Alcohol Content; Table S2 Results of Different Spectral Preprocessing Methods for Original Wort Concentration; Table S3 Results of Different Spectral Preprocessing Methods for Authenticity Identification; Table S4 Results of Different Spectral Preprocessing Methods in MTL Strategies; Table S5 Craft Beer and Industrial Beer Sample Information; Table S6 Blended beer sample information; Table S7 CNN-MHA Parameter Optimization Range; Table S8 LSTM-MHA Parameter Optimization Range; Table S9 CNN-LSTM-MHA Parameter Optimization Range.
