# Supplementary material for: Key Indicator Detection and Authenticity Identification of Beer Based on Near-Infrared Spectroscopy Combined with Multi-Task Feature Extraction
Source: Molecules. 2026 Mar 26;31(7):1083. doi: 10.3390/molecules31071083 (PMC13074725; doi:10.3390/molecules31071083)
Supplement: Supplementary file 1 [file molecules-31-01083-s001.zip › Supplementary Material_v8.pdf]

Table S1 Results of Different Spectral Preprocessing Methods for Alcohol Content

Preprocessing methods	R_{cv}^2	RMSECV	RPDCV	LVs
N/A	0.939	0.358	4.685	10
SG	0.939	0.358	4.682	10
MSC	0.931	0.379	4.447	13
SNV	0.929	0.386	4.374	13
WD	0.939	0.357	4.702	10
FTD	0.939	0.358	4.699	10
SG+MSC	0.925	0.389	4.432	12
SG+SNV	0.929	0.380	4.496	13
SG+WD	0.939	0.359	4.676	10
SG+FTD	0.939	0.358	4.700	10
MSC+SNV	0.929	0.383	4.384	13
MSC+WD	0.935	0.366	4.630	13
MSC+FTD	0.920	0.402	4.309	9
SNV+WD	0.936	0.363	4.649	14
SNV+FTD	0.921	0.401	4.317	9
WD+FTD	0.939	0.358	4.676	10

R_{cv}^2、RMSECV and RPDCV represent R^2、RMSE and RPD of cross-validation, respectively. LVs are the principal components extracted from PLSR. The optimal number of LVs is determined by the minimum PRESS value obtained during cross-validation.

Table S2 Results of Different Spectral Preprocessing Methods for Original Wort Concentration

Preprocessing methods	R_{cv}^2	RMSECV	RPDCV	LVs
N/A	0.682	1.208	2.129	12
SG	0.686	1.206	2.141	12
MSC	0.713	1.160	2.217	10
SNV	0.780	1.016	2.506	11
WD	0.747	1.077	2.382	13
FTD	0.670	1.227	2.119	12
SG+MSC	0.710	1.146	2.256	12
SG+SNV	0.757	1.065	2.433	11
SG+WD	0.687	1.197	2.160	12
SG+FTD	0.676	1.220	2.118	12
MSC+SNV	0.775	1.019	2.489	11
MSC+WD	0.687	1.178	2.217	10
MSC+FTD	0.681	1.172	2.254	14
SNV+WD	0.721	1.119	2.319	10
SNV+FTD	0.720	1.120	2.336	12
WD+FTD	0.710	1.162	2.220	12

R_{cv}^2 、RMSECV and RPDCV represent R^2 、RMSE and RPD of cross-validation, respectively. The optimal number of LVs is determined by the minimum PRESS value obtained during cross-validation.

Table S3 Results of Different Spectral Preprocessing Methods for Authenticity Identification

Preprocessing methods	ACCCV(%)	CVER(%)	PrecisionCV(%)	RecallCV(%)	LVs
N/A	99.440	0.560	99.380	99.490	15
SG	99.180	0.820	99.160	99.260	15
MSC	99.340	0.660	99.350	99.370	18
SNV	99.320	0.680	99.330	99.350	19
WD	99.570	0.430	99.610	99.600	16
FTD	99.390	0.610	99.390	99.460	18
SG+MSC	99.130	0.870	99.180	99.170	17
SG+SNV	99.110	0.890	99.170	99.150	17
SG+WD	99.400	0.600	99.410	99.440	15
SG+FTD	99.380	0.620	99.380	99.450	17
MSC+SNV	99.110	0.890	99.180	99.040	12
MSC+WD	99.120	0.880	99.180	99.160	18
MSC+FTD	99.080	0.920	99.140	99.020	16
SNV+WD	99.240	0.760	99.280	99.270	16
SNV+FTD	99.150	0.850	99.210	99.080	15
WD+FTD	99.580	0.420	99.640	99.620	19

ACCCV, CVER, PrecisionCV, and RecallCV represent the accuracy, error rate, precision, and recall rate in cross-validation, respectively. LVs denote the principal components extracted in PLS-DA. The optimal number is determined by the minimum error rate obtained through cross-validation.

Table S4 Results of Different Spectral Preprocessing Methods in MTL Strategies

Preprocessing methods	Alcohol Content RMSECV	Original Wort Concentration RMSECV	Classification CVER (%)	MCPS
N/A	0.358	1.208	0.560	1.175
SG	0.358	1.206	0.820	1.381
MSC	0.379	1.160	0.660	1.258
SNV	0.386	1.016	0.680	1.233
WD	0.357	1.077	0.430	1.028
FTD	0.358	1.227	0.610	1.221
SG+MSC	0.389	1.146	0.870	1.430
SG+SNV	0.380	1.065	0.890	1.411
SG+WD	0.359	1.197	0.600	1.204
SG+FTD	0.358	1.220	0.620	1.227
MSC+SNV	0.383	1.019	0.890	1.398
MSC+WD	0.366	1.178	0.880	1.427
MSC+FTD	0.402	1.172	0.920	1.490
SNV+WD	0.363	1.119	0.760	1.309
SNV+FTD	0.401	1.120	0.850	1.417
WD+FTD	0.358	1.162	0.420	1.049

Alcohol Content RMSECV and Original Wort Concentration RMSECV represent the root mean square error of cross-validation for alcohol content and original wort concentration prediction, respectively. Classification CVER represents the error rate for the identification task in cross-validation. MCPS denotes the Multi-task Comprehensive Performance Score.

Table S5 Craft Beer and Industrial Beer Sample Information

Serial number	Category	Sample name	Place of Origin	Production Date	Alcohol content (%vol)[a]	Original wort concentration (°P)[a]
1	Craft Beer	Harbin Snow Bear	China	2025/7/10	≥4	11
2	Craft Beer	Feiyero White Beer	China	2025/7/15	≥3.9	11
3	Craft Beer	Tsingtao 1819	China	2025/7/22	≥3.3	9
4	Craft Beer	Cool Little Beer	China	2025/8/9	≥3.6	10
5	Craft Beer	Kersen	China	2025/7/18	≥4.8	12
6	Craft Beer	Polar Brothers	China	2025/7/12	≥3.7	10.5
7	Craft Beer	Caesar Orchard	China	2025/7/20	≥4	11
8	Craft Beer	Caesar King	China	2025/7/14	≥4	11
9	Craft Beer	Crazy Tiger	China	2025/7/21	≥3.6	10
10	Craft Beer	Demaiguan (German Crown)	China	2025/7/11	≥3.6	10
11	Craft Beer	Aigenburg	Germany	2025/7/19	≥4.1	11
12	Craft Beer	Oujia Viper	China	2025/7/16	≥5	12
13	Craft Beer	Snow Bear White Beer	China	2025/7/23	≥3.3	10
14	Craft Beer	Genting Felder	China	2025/7/13	≥3.3	10
15	Craft Beer	Maikelei	China	2025/7/17	≥12	24
16	Craft Beer	Ledingburg Pilsner	China	2025/8/8	≥4.2	11
17	Craft Beer	Lost Coast	United States	2025/7/12	≥8.7	18
18	Craft Beer	Hiccup Beaver (Highway)	United States	2025/7/9	≥7.3	17
19	Craft Beer	Lager Barley	China	2025/7/15	≥4.3	11.5
20	Craft Beer	Hiccup Beaver (Phantom Bride)	China	2025/8/5	≥7.1	15
21	Craft Beer	Zhadan Laopi	China	2025/7/12	≥3.3	9
22	Craft Beer	Fansizhe White Beer	China	2025/7/21	≥4.1	11
23	Craft Beer	Six Swans	China	2025/7/14	≥4.5	13

24	Industrial Beer	Blue Lion Extra Dry	China	2025/7/20	≥4.3	11
25	Industrial Beer	Tsingtao Ice Pure	China	2025/8/3	≥4.0	11
26	Industrial Beer	Harbin Ha Super Fresh	China	2025/7/18	≥3.6	10
27	Industrial Beer	Snowflake Ice Cool	China	2025/7/11	≥3.9	10.3
28	Industrial Beer	Snowflake Pure Draft	China	2025/7/16	≥3.2	8
29	Industrial Beer	Harbin Pure Refresh	China	2025/7/23	≥3.3	10
30	Industrial Beer	1664 White Beer	France	2025/7/11	≥4.8	11.8
31	Industrial Beer	Wusu	China	2025/7/17	≥4.0	11
32	Industrial Beer	Harbin 1900	China	2025/7/8	≥3.6	10
33	Industrial Beer	Tsingtao Pure Draft	China	2025/7/22	≥3.1	8
34	Industrial Beer	Budweiser Pure Draft	China	2025/8/2	≥3.1	8
35	Industrial Beer	Harbin Wheat King	China	2025/7/15	≥3.6	10
36	Industrial Beer	Laoshan Refresh	China	2025/7/19	≥3.1	8

[a] The specified value marked on the beer bottle.

Table S6 Blended beer sample information

Serial number	Auxiliary materials(g)	Ethanol(ml)	Distilled water(ml)	Alcohol content(%vol)	Original wort concentration(°P)
1	23.81	6.32	169.87	3	8
2	23.81	8.42	167.77	4	8
3	23.81	10.53	165.66	5	8
4	23.81	14.74	161.45	7	8
5	26.75	6.32	166.93	3	9
6	26.75	8.42	164.83	4	9
7	26.75	10.53	162.72	5	9
8	26.75	14.74	158.51	7	9
9	29.69	6.32	163.99	3	10
10	29.69	8.42	161.89	4	10
11	29.69	10.53	159.78	5	10
12	29.69	14.74	155.57	7	10
13	32.63	6.32	161.06	3	11
14	32.63	8.42	158.95	4	11
15	32.63	10.53	156.84	5	11
16	32.63	14.74	152.63	7	11
17	35.57	6.32	158.12	3	12
18	35.57	8.42	156.01	4	12
19	35.57	10.53	153.91	5	12
20	35.57	14.74	149.7	7	12

Auxiliary materials refers to the volume of sodium bicarbonate, beer flavorings, and anhydrous citric acid.

Table S7 CNN-MHA Parameter Optimization Range

Target Task	Parameter	Range
Alcohol content<br>Original wort<br>Classification and Identification<br>(STL)	F_Small	[2, 5]
	F_Mid	[6, 12]
	F_Large	[13, 30]
	NumFilters	[8, 32]
	FeatureDimension	[30, 200]
	InitialLearnRate	[0.001, 0.1]
	L2Regularization	[1e-10, 1e-2]
Shared Features<br>(MTL)	F_Small	[2, 5]
	F_Mid	[6, 12]
	F_Large	[13, 30]
	NumFilters	[8, 32]
	FeatureDimension	[30, 200]
	InitialLearnRate	[0.001, 0.1]
	L2Regularization	[1e-10, 1e-2]
	Alcohol Content Weighting	[0.1, 0.9]
	Original Wort Weight	[0.1, 0.9]
	Classification Discrimination Weight	[0.1, 0.9]

F_Small, F_Mid, F_Large, NumFilters, NumResponses, InitialLearnRate, Alcohol Content Weighting, Original Wort Weight, Classification Discrimination Weight, and L2Regularization represent the small kernel size, medium kernel size, large kernel size, number of filters, feature dimension of the fully connected layer, initial learning rate, alcohol content weight, original wort concentration weight, classification discrimination weight, and L2 regularization coefficient, respectively.

Table S8 LSTM-MHA Parameter Optimization Range

Target Task	Parameter	Range
Alcohol content<br>Original wort<br>Classification and Identification<br>(STL)	LstmHiddenUnits	[30, 100]
	FeatureDimension	[20, 80]
	InitialLearnRate	[1e-3, 5e-2]
	LearnRateDropFactor	[0.02, 0.9]
	L2Regularization	[1e-10, 1e-2]
	DropoutRate	[0.1, 0.5]
Shared Features<br>(MTL)	LstmHiddenUnits	[30, 100]
	FeatureDimension	[20, 80]
	InitialLearnRate	[1e-3, 5e-2]
	LearnRateDropFactor	[0.02, 0.9]
	L2Regularization	[1e-10, 1e-2]
	DropoutRate	[0.1, 0.5]
	Alcohol Content Weighting	[0.1, 0.9]
	Original Wort Weight	[0.1, 0.9]
	Classification Discrimination Weight	[0.1, 0.9]

LstmHiddenUnits, FeatureDimension, InitialLearnRate, LearnRateDropFactor, L2Regularization, Alcohol Content Weighting, Original Wort Weight, Classification Discrimination Weight, and DropoutRate represent the number of LSTM hidden units, the feature dimension of the fully connected layer, the initial learning rate, the learning rate drop factor, the L2 regularization coefficient, the alcohol content weight, the original wort concentration weight, the classification discrimination weight, and the dropout rate, respectively.

Table S9 CNN-LSTM-MHA Parameter Optimization Range

Target Task	Parameter	Range
Alcohol content<br>Original wort<br>Classification and Identification<br>(STL)	F_Small	[2, 5]
	F_Mid	[6, 12]
	F_Large	[13, 30]
	NumFilters	[8, 32]
	LstmHiddenUnits	[30, 100]
	FeatureDimension	[20, 80]
	InitialLearnRate	[1e-3, 5e-2]
	LearnRateDropFactor	[0.02, 0.9]
	L2Regularization	[1e-10, 1e-2]
	DropoutRate	[0.1, 0.5]
Shared Features<br>(MTL)	F_Small	[2, 5]
	F_Mid	[6, 12]
	F_Large	[13, 30]
	NumFilters	[8, 32]
	LstmHiddenUnits	[30, 100]
	FeatureDimension	[20, 80]
	InitialLearnRate	[1e-3, 5e-2]
	LearnRateDropFactor	[0.02, 0.9]
	L2Regularization	[1e-10, 1e-2]
	DropoutRate	[0.1, 0.5]
	Alcohol Content Weighting	[0.1, 0.9]
	Original Wort Weight	[0.1, 0.9]
	Classification Discrimination Weight	[0.1, 0.9]

F_Small, F_Mid, F_Large, NumFilters, LstmHiddenUnits, FeatureDimension, InitialLearnRate, LearnRateDropFactor, L2Regularization, Alcohol Content Weighting, Original Wort Weight, Classification Discrimination Weight, and DropoutRate represent the small kernel size, medium kernel size, large kernel size, number of filters, number of LSTM hidden units, feature dimension of the fully connected layer, initial learning rate, learning rate drop factor, L2 regularization coefficient, alcohol content weight, original wort concentration weight, classification discrimination weight, and dropout rate, respectively.
